# Reproductive and Developmental Toxicity of Microcystin-LR in Mammals

**DOI:** 10.3390/toxics14080734

**Published:** 2026-08-21

**Authors:** Youngran Ku, Sooyeon Park, Changwon Yang

**Affiliations:** Department of Science Education, Ewha Womans University, Seoul 03760, Republic of Korea; fks3615@ewha.ac.kr (Y.K.); spark01@ewha.ac.kr (S.P.)

**Keywords:** microcystin-LR, reproductive toxicity, endocrine disruption, cyanotoxins, developmental programming

## Abstract

Cyanobacterial harmful algal blooms have increased globally, likely in part due to climate change and anthropogenic eutrophication, elevating exposure of humans and other terrestrial and aquatic animals to cyanotoxins through drinking water, food chains, and recreational activities. Microcystin-LR (MC-LR), the most prevalent microcystin congener, is well known for its hepatotoxicity, yet accumulating evidence indicates that it also exerts reproductive and developmental toxicity in mammals. This review summarizes recent experimental and epidemiological studies indicating that MC-LR disrupts reproductive function through direct cellular injury to germ and somatic cells, dysregulation of gonadal steroidogenesis, and impairment of the hypothalamic-pituitary-gonadal (HPG) axis. In males, MC-LR disrupts spermatogenesis and sperm quality, while in females it impairs oocyte competence, uterine receptivity, and placental function, leading to adverse pregnancy outcomes. Importantly, exposure during critical developmental windows can influence developmental trajectories and contribute to long-term, multi-organ dysfunction in offspring via endocrine imbalance and epigenetic reprogramming. Mechanistically, MC-LR toxicity involves convergent oxidative stress, mitochondrial dysfunction, inflammatory signaling, DNA damage, and chromatin remodeling. Collectively, these findings indicate that reproductive and developmental toxicity should be considered in assessments of the health risks associated with MC-LR and support the incorporation of reproductive endpoints into human health and ecological risk assessment frameworks.

## 1. Introduction

Over recent decades, cyanobacterial harmful algal blooms have intensified in both frequency and magnitude worldwide, driven largely by climate change-associated increases in global temperatures and by anthropogenic eutrophication resulting from agricultural runoff and rapid urbanization [1]. As ubiquitous microbial inhabitants of fresh, brackish, and marine waters, cyanobacteria are capable of undergoing periods of massive population growth, leading to the formation of extensive and often toxic algal blooms [2]. Based on 37 years of satellite observations from 1982 to 2018, 66% (466 of 708) of the world’s lakes with detectable algal blooms exhibited increasing trends in either bloom frequency or bloom area [3]. A principal component regression model applied under shared socioeconomic pathway (SSP) scenarios projected that the number of lakes showing an increase in fractional floating algal cover would rise from 1134 under SSP1-2.6 to 1495 under SSP2-4.5 and 1526 under SSP4-6.0, suggesting that algal bloom conditions may intensify under stronger climate forcing [4]. First identified through animal fatalities in the late 1870s, the danger of cyanobacteria to ecosystem and public health has since been reinforced by ongoing documentation of human toxicity [5]. Human exposure to aquatic cyanobacteria and their toxins can occur through multiple pathways, including the consumption of contaminated drinking water, dermal contact and accidental ingestion during recreational activities, and dietary intake of foods such as spray-irrigated vegetables or aquatic organisms where toxins have bioaccumulated [6]. Although quantitative evidence regarding the relative contribution of human cyanotoxin exposure pathways is lacking, ingestion of cyanotoxin-contaminated drinking water is considered the leading pathway of human exposure [7].

Microcystins (MCs) are the most globally prevalent class of cyanotoxins, synthesized by a diverse array of cyanobacterial genera including *Microcystis*, *Anabaena* (*Dolichospermum*), *Planktothrix*, and the terrestrial genus *Hapalosiphon* [8,9]. The nomenclature of MCs is derived from *Microcystis aeruginosa*, the cyanobacterial species from which these toxins were first isolated [10]. MCs pose a considerable health risk to humans, livestock, and wildlife, attributable to diverse environmental exposure pathways [11,12].

Epidemiological investigations across multiple geographical regions have consistently reported a broad spectrum of adverse health outcomes associated with human exposure to MCs [13,14]. During active cyanobacterial blooms in two California reservoirs, microcystin concentrations ranged from 10 to 500 µg/L, indicating potential exposure concerns for terrestrial mammals dependent on these water sources [15]. Other bloom-season reports include seasonal concentrations of 6.69–15.6 μg/L in Lake Taihu, China [16,17]. Field investigations, such as those conducted at the Ain Zada dam, have reported substantial microcystin contamination, with concentrations reaching 19.6 mg/L in raw water and remaining at 6.3 mg/L after drinking-water treatment, indicating potential public health concerns [18]. These values are presented as illustrative exposure contexts and should not be interpreted as directly comparable because the studies differ in analyte definition, sampling design, analytical method, and water-treatment context.

Recent environmental syntheses suggest that climate-related changes in bloom timing and duration may prolong cyanotoxin exposure seasons and increase the potential for recurrent or near-year-round exposure in some regions [19,20]. Consistent with this shift toward sustained environmental exposure, earlier investigations have documented bioaccumulation of MCs within the gonadal tissues of lower aquatic organisms, including mollusks and crustaceans [21]. Such bioaccumulation raises concern that contaminated aquatic organisms may contribute to dietary exposure in higher-order consumers, although direct evidence for trophic transfer to mammals remains limited [6,21].

MCs are cyclic heptapeptides containing two variable L-amino acids and the conserved Adda (3-amino-9-methoxy-2,6,8-trimethyl-10-phenyldeca-4,6-dienoic acid) moiety (Figure S1) [22]. More than 300 congeners have been identified, with MC-LR among the most prevalent and extensively studied forms [10]. Its cyclic structure contributes to resistance to heat, hydrolysis, and oxidation, and conventional water treatment may not completely remove dissolved MC-LR [7,10]. This persistence facilitates long-term environmental exposure and increases the likelihood of human and wildlife intake through drinking water and the food chain. Notably, MC-LR has been detected in human serum, with concentrations ranging from 0.10 to 0.64 μg/L in fishermen chronically exposed to cyanobacteria-contaminated water bodies, providing direct evidence of internal exposure in occupationally at-risk populations [23]. Beyond serum, total MCs have also been detected in human semen, although the immunoassay used did not distinguish MC-LR from other microcystin congeners [24]. More recently, MC-LR was quantified in human placental tissues from patients with preeclampsia or fetal growth restriction [25].

Due to its hydrophilic nature, MC-LR cannot passively diffuse across cell membranes and instead relies on carrier-mediated active transport, primarily facilitated by organic anion transporting polypeptides (OATPs) [26]. It is postulated that the liver’s high susceptibility to MCs stems from the presence of specific transport systems that efficiently concentrate the toxin within hepatocytes [27]. By cataloging the diverse health effects across various organs, previous reviews support the premise that MCs are not solely hepatotoxic but are systemic toxins. Massey and colleagues addressed the health effects of MCs on the intestines, brain, kidneys, lungs, and reproductive systems of animals and humans [11]. McLellan and colleagues have also extensively reported on the toxic mechanisms that MCs may exhibit in mammalian tissues, including protein phosphorylation and ROS formation [28]. Arman and Clarke also discussed the toxicokinetics of MC-LR and provided preclinical evidence supporting the systemic toxicity of MC-LR [29]. While the reproductive toxicity of MCs has been comprehensively reviewed previously, a substantial body of research has since accumulated, particularly concerning sex-specific impacts and transgenerational toxicity in mammals [30]. Another review centered on male reproductive toxicity has established a solid mechanistic foundation for understanding MC-LR-induced gonadal injury [31]. However, the specific mechanisms, critical windows, and developmental consequences of MC-LR exposure have not been sufficiently integrated within a reproductive and developmental biology framework. Previous reviews established the reproductive toxicity of MCs across species and summarized major toxic mechanisms. However, female reproductive effects, placental injury, critical exposure windows, and long-term offspring outcomes in mammals have not been fully integrated. This review addresses these gaps by linking sex-specific reproductive toxicity and developmental outcomes with HPG axis disruption and emerging molecular mechanisms. Although acute toxicity benchmarks such as LD_50_ values highlight the potent lethality of MC-LR, growing evidence indicates that sublethal and chronic exposures can exert profound reproductive and developmental effects that are not captured by traditional toxicity endpoints. This review, therefore, aims to provide a more in-depth and systematic analysis of the reproductive and developmental toxicity of MC-LR based on recent findings.

PubMed and Google Scholar were searched from database inception to 31 January 2026 using the Boolean search string “microcystin” AND “reproductive.” Supplementary searches combined “microcystin” with “developmental,” “placental,” “prenatal,” or “perinatal.” After duplicate removal, 297 records were screened. Original studies examining the reproductive or developmental effects of MCs in mammalian systems were included. Reviews, non-mammalian studies, and studies without relevant outcomes were excluded from the primary evidence synthesis. Review articles were used for background information and to contextualize previous findings. Only English-language publications were included. A total of 111 studies met these criteria. The study selection process is shown in Figure S2. This focused approach enables a coherent synthesis of current mechanistic evidence while highlighting critical knowledge gaps that warrant further investigation.

## 2. Male Reproductive Toxicity of MC-LR

### 2.1. Testicular Toxicity

As summarized in earlier reviews, the testis has been established as a critical target organ for MC-LR, where the toxin crosses the blood-testis barrier and induces molecular damage primarily through oxidative stress and genotoxicity [32]. Recent studies further reinforce the vulnerability of the male reproductive system to MC-LR exposure (Table 1). Confirming direct exposure, MC-LR was detected in the testes in male rats following acute intraperitoneal administration (300 µg/kg/day for 6 days), with immunofluorescence showing localization primarily within the seminiferous tubules [33]. MC-LR is mainly localized in spermatogonia and Sertoli cells, with little detection in the Leydig cell-rich interstitial tissue. This suggests that the seminiferous epithelium is the primary direct target, whereas Leydig cell dysfunction may arise mainly through HPG axis disruption and local inflammatory responses. Consistent with this cellular distribution and tissue accumulation, subsequent in vivo studies have provided direct morphological evidence of testicular injury following MC exposure.

A single intraperitoneal injection of microcystin extracts (12.5 µg/kg) induces significant, albeit transient, ultrastructural damage in the testes of male rabbits [45]. Acute exposure to MC-LR induces dose- and time-dependent apoptosis in mouse testicular cells, primarily localized within the seminiferous tubules [46]. Significant increases in apoptotic cells were observed following 4 days of intraperitoneal administration at doses of 7.5 µg/kg/day and higher. In male mice, intraperitoneal administration of MC-LR (10–40 μg/kg/day for 2 weeks) significantly reduced the gonadosomatic index and induced marked histopathological damage in the testes, including disorganized seminiferous tubules, spermatogenic cell shedding, and reduced sperm density [34]. A separate two-week study showed that intraperitoneal administration of MC-LR at doses of 15 and 30 µg/kg daily for 14 days induced significant testicular atrophy and histological lesions, including the extensive loss of germ cells and the formation of multinucleated giant cells within seminiferous tubules [47]. In young male rats, acute exposure (1 week) to MC-LR causes significant structural damage to the testes, including reduced testicular weight, decreased seminiferous tubule diameter, and disruption of the spermatogenic epithelium, particularly evident at a dose of 100 µg/kg/day [48]. Extending the exposure to MC-LR for 4 weeks in male rats leads to a significant reduction in testis weight at a dose of 15 µg/kg/day [49]. Histological analysis of the testes revealed severe damage, including atrophy and obstruction of the seminiferous tubules. Such profound structural disruption of the seminiferous tubules raises the possibility that MC-LR exposure impairs the integrity of the blood-testis barrier (BTB), a critical protective interface within the mammalian testis. Intraperitoneal administration of 7.5–30 µg/kg MC-LR for 1 week disrupted the integrity of the BTB, leading to the infiltration of immune cells and the generation of autoantibodies against germ cells [50]. This physiological disruption is characterized by the internalization and dramatic downregulation of key tight junction proteins, including occludin and ZO-1, which are essential for maintaining the BTB [51].

Such disruption of testicular architecture and barrier integrity provides a critical mechanistic framework for understanding how repeated or chronic MC-LR exposure can progressively impair spermatogenesis. This conceptual link is supported by experimental evidence from long-term in vivo exposure rat models, in which 50-day MC-LR administration induces significant testicular toxicity. At 10 µg/kg/day, this manifests as a decreased testis index, widened intratubular spaces, and severe ultrastructural damage, including mitochondrial swelling, cell membrane blebbing, and deformed nuclei [52]. Under chronic oral exposure conditions, MC-LR induces significant and cumulative testicular damage in male mice. After 6 months of exposure, structural damage to the spermatogenic epithelium is observed at a dose of 10 µg/L, while significant increases in testicular cell apoptosis are seen at doses as low as 3.2 µg/L [53]. Consistent with this finding, long-term exposure to environmentally relevant concentrations of MC-LR (1–120 µg/L) via drinking water for 6–12 months induced marked histopathological alterations in mouse testes, including disorganized seminiferous tubules, germ cell exfoliation, and structural degeneration [35]. Together, these findings suggest that the progressive testicular damage induced by MC-LR is not merely a consequence of generalized tissue injury but is likely driven by dysfunction of specific somatic cell populations within the seminiferous epithelium.

The integrity of Sertoli cells is crucial for spermatogenesis, as toxicants that impair their function also diminish their capacity to provide the essential nutritional and physical support required by developing germ cells [54]. MC-LR directly targets Sertoli cells, reducing cell viability and inducing apoptosis at 10 µg/mL [55]. This concentration greatly exceeds reported environmental levels and is mainly relevant to mechanistic evaluation rather than environmental risk assessment. Similarly, exposure to 8, 16, and 32 µg/mL MC-LR for 24 h resulted in a dose-dependent reduction in cell viability and a significant induction of apoptosis in primary rat Sertoli cells [56]. The similarly high concentrations used in these studies should therefore be interpreted as experimental conditions for identifying cellular injury pathways. Importantly, adverse effects on Sertoli cell function are also evident at substantially lower concentrations. In mouse Sertoli cells, exposure to 500 nM MC-LR for 24 h significantly reduced cell viability and decreased the expression of key tight junction proteins such as ZO-1 and occludin, potentially compromising the integrity of the BTB [57]. Studies on primary rat Sertoli cells confirm that MC-LR is directly cytotoxic, significantly decreasing cell viability and compromising membrane integrity at concentrations of 50 nM and 500 nM after 24 to 48 h of exposure [58]. These concentrations correspond to approximately 50–500 µg/L and overlap with levels reported during intense bloom events. However, they exceed typical chronic low-level exposure, and direct extrapolation to internal testicular exposure remains limited. Moreover, exposure of mouse Sertoli cells to MC-LR at concentrations of 7.5, 15, and 30 nM for 24 h induces a dose-dependent increase in apoptosis, thereby compromising the structural and nutritional support essential for spermatogenesis [36]. In bovine models, treatment with 40–80 µg/L MC-LR for 48 h significantly reduces Sertoli cell viability and induces nuclear morphological damage, including chromatin condensation and atrophy [59]. This direct toxicity towards the supportive Sertoli cells further explains the disruption of spermatogenesis observed in vivo.

Leydig cells are responsible for synthesizing the androgens that are essential for male development and the maintenance of spermatogenesis [60]. Accordingly, chemical-induced injury to these cells represents a direct mechanism that can disrupt spermatogenesis and ultimately impair male fertility. However, compared with the extensive evidence describing direct MC-LR toxicity toward Sertoli cells, the effects of MC-LR on Leydig cells appear to be less well characterized and are largely inferred from indirect or histopathological observations. Chronic exposure to MC-LR induces significant structural changes in the testicular interstitium, specifically a marked reduction in the Leydig cell population and a concomitant increase in the number of testicular macrophages [61]. Overall, MC-LR–induced testicular toxicity predominantly affects the seminiferous epithelium, while Leydig cell dysfunction likely plays a secondary and indirect role. Thus, Leydig cell toxicity remains an important knowledge gap. Further studies are needed to distinguish direct cellular effects from secondary changes driven by local inflammation or HPG axis disruption.

### 2.2. Disruption of Spermatogenesis and Sperm Function

Human epidemiological evidence supports the relevance of MC exposure to impaired sperm function. In a cross-sectional study of 1715 men from cyanobacterial bloom-prone regions of China, higher semen MC levels were associated with reduced sperm count and motility, impaired velocity, and increased abnormal morphology [24]. In a subsequent analysis of 837 participants from the same cohort, MCs were detected in 88.6% of semen samples at a mean concentration of 0.16 μg/L using an immunoassay that did not distinguish individual congeners. Increasing ln-transformed MC levels were associated with lower total motility (β = −4.387), progressive motility (β = −4.428), and curvilinear velocity (β = −1.481) [62]. These associations were accompanied by reduced levels of epididymal and prostatic biochemical markers, suggesting that accessory reproductive tissues may partly contribute to impaired sperm motility. Spermatogenesis, the intricate process of sperm production, is initiated by the proliferation of spermatogonia within the testicular environment. Acute exposure to MCs causes significant testicular damage in male rats by inducing a rapid and marked increase in the apoptosis of testicular germ cells [63]. This programmed cell death was observed to peak approximately 6 h after exposure. Consistent with this germ-cell injury, a dose-dependent decline in sperm quality was observed in mice treated with 7.5, 15, and 30 µg/kg MC-LR for 1 week, where epididymal sperm concentration and motility were significantly reduced while sperm abnormality rates increased [50]. Notably, pubertal exposure to 2 µg/kg MC-LR via intraperitoneal injection for 4 weeks in mice significantly decreases epididymal sperm concentration and inhibits spermatogonia proliferation [37]. Long-term MC-LR exposure (≥15 μg/L for 6 months) significantly reduced epididymal sperm counts and increased the proportion of morphologically abnormal spermatozoa in mice [64]. Similarly, long-term MC-LR exposure markedly disrupted spermiogenesis, as evidenced by selective loss of spermatocytes and round spermatids and a high prevalence of abnormal sperm head morphology associated with defective acrosome formation [38].

MC-LR directly targets spermatogonia in vitro, significantly reducing cell viability and inducing apoptosis after 6 h of exposure at concentrations of 5 nM and higher [65]. An in vitro study using the mouse spermatogonia cell line further demonstrated that exposure to 500 nM MC-LR for 24 h significantly reduced cell viability and induced apoptosis [66]. MC-LR targets spermatogonia by entering the cells via OATPs, where it triggers diverse signaling cascades leading to germ cell apoptosis [65]. These findings indicate that MC-LR toxicity begins at the earliest stages of germ cell development, potentially contributing to the observed decline in overall sperm quality and quantity.

In line with these findings, multiple in vivo studies have reported compromised sperm function following MC-LR exposure. While sperm concentration and the rate of morphological abnormalities were not significantly affected, sperm viability and motility were both significantly reduced in treated mice compared with controls [67]. In another study, MC-LR caused a decrease in sperm motility, a reduction in overall sperm concentration, and a dose-dependent increase in the rate of sperm abnormalities [49]. Long-term, low-dose ingestion of MC-LR leads to a significant, dose- and time-dependent decline in sperm quality [53]. After 6 months, exposure to 3.2 and 10 µg/L MC-LR in drinking water results in reduced sperm counts and motility, as well as a significantly higher rate of sperm abnormalities in male mice. Collectively, experimental and epidemiological evidence indicates that MC-LR exposure is associated with impaired sperm quality, and animal studies show that these effects occur under long-term, low-dose exposure.

### 2.3. Prostate Pathology and Malignant Transformation

Functioning as a key reproductive organ, the prostate produces prostatic fluid, which constitutes a vital portion of the seminal volume and is essential for successful male reproduction. In contrast to the extensive literature describing MC-LR-induced dysfunction of germ cells and testicular cells, evidence regarding prostatic toxicity has largely focused on pathological remodeling and carcinogenic outcomes, with less attention to direct impairment of reproductive function. Emerging evidence suggests that environmental contaminants may represent an underrecognized contributor to prostate carcinogenesis [68]. Epidemiological evidence indicates that environmental exposure to MC-LR is associated not only with reproductive dysfunction but also with increased risk and poorer prognosis of prostate cancer. In a case–control study involving 238 prostate cancer patients and matched controls, higher serum MC-LR levels (≥0.38 ng/mL) were associated with a significantly increased risk of prostate cancer [69]. Consistent with these observations, chronic oral exposure to 10–30 μg/L MC-LR via drinking water for 3 to 6 months induces focal epithelial hyperplasia and stroma overgrowth in the prostate of adult mice, leading to a significantly increased prostate index [70]. Similarly, chronic exposure to MC-LR via drinking water at concentrations ranging from 1 to 120 µg/L for 6 months induced prostatic intraepithelial neoplasia and microinvasion in mice, suggesting a direct link between long-term cyanotoxin ingestion and prostate carcinogenesis [41]. At the cellular level, MC-LR exposure (1–5 μM for 24 h) enhances the invasive capacity of human prostate cancer DU145 cells without affecting cell viability, indicating a role in promoting malignant progression without overt cytotoxicity [42]. Collectively, these findings indicate that MC-LR-induced prostatic toxicity is characterized predominantly by tumor-promoting and carcinogenic effects, distinguishing the prostate from other male reproductive organs in which the primary toxicological outcome is functional impairment.

### 2.4. Disruption of Male Reproductive Hormone Homeostasis

Beyond direct structural and cellular damage within the male reproductive organs, accumulating evidence indicates that MC-LR also perturbs systemic endocrine regulation. Given that spermatogenesis, sperm maturation, and accessory gland function are tightly governed by hormonal signaling, disruption of endocrine homeostasis represents a critical mechanism underlying MC-LR-induced male reproductive toxicity. MC-LR has been shown to disrupt the hypothalamic-pituitary-gonadal (HPG) axis in mammalian models, thereby contributing to impaired spermatogenesis and reduced male fertility [71]. Acute intraperitoneal administration of MC-LR to male rats (45, 67.5, or 90 μg/kg) resulted in marked disruption of the HPG axis within 24 h [43]. Comparative reviews have highlighted that the male reproductive system’s response to MC-LR, including HPG axis disruption, shows consistent pathological patterns across both aquatic and mammalian models [72]. These conserved responses point to disruption of central neuroendocrine regulation as a shared mechanistic basis across species. The synthesis and secretion of the pituitary gonadotropins, LH and FSH, are strictly orchestrated by the pulsatile release of gonadotropin-releasing hormone (GnRH) from the hypothalamus. Chronic oral exposure to 7.5, 15, or 30 µg/L MC-LR via drinking water for 6 months in male mice significantly reduces serum levels of both GnRH [73]. Consistent with this finding, exposure to 30 µg/kg/day for 6 weeks triggers a complex hormonal shift in the male reproductive axis, characterized by a significant reduction in serum GnRH levels [44]. Even in a short-term exposure model, intraperitoneal injection of 15, 20, or 30 µg/kg MC-LR for 7 consecutive days significantly reduced GnRH expression in the hypothalamus and serum testosterone levels in male mice [74].

**Figure 1:**
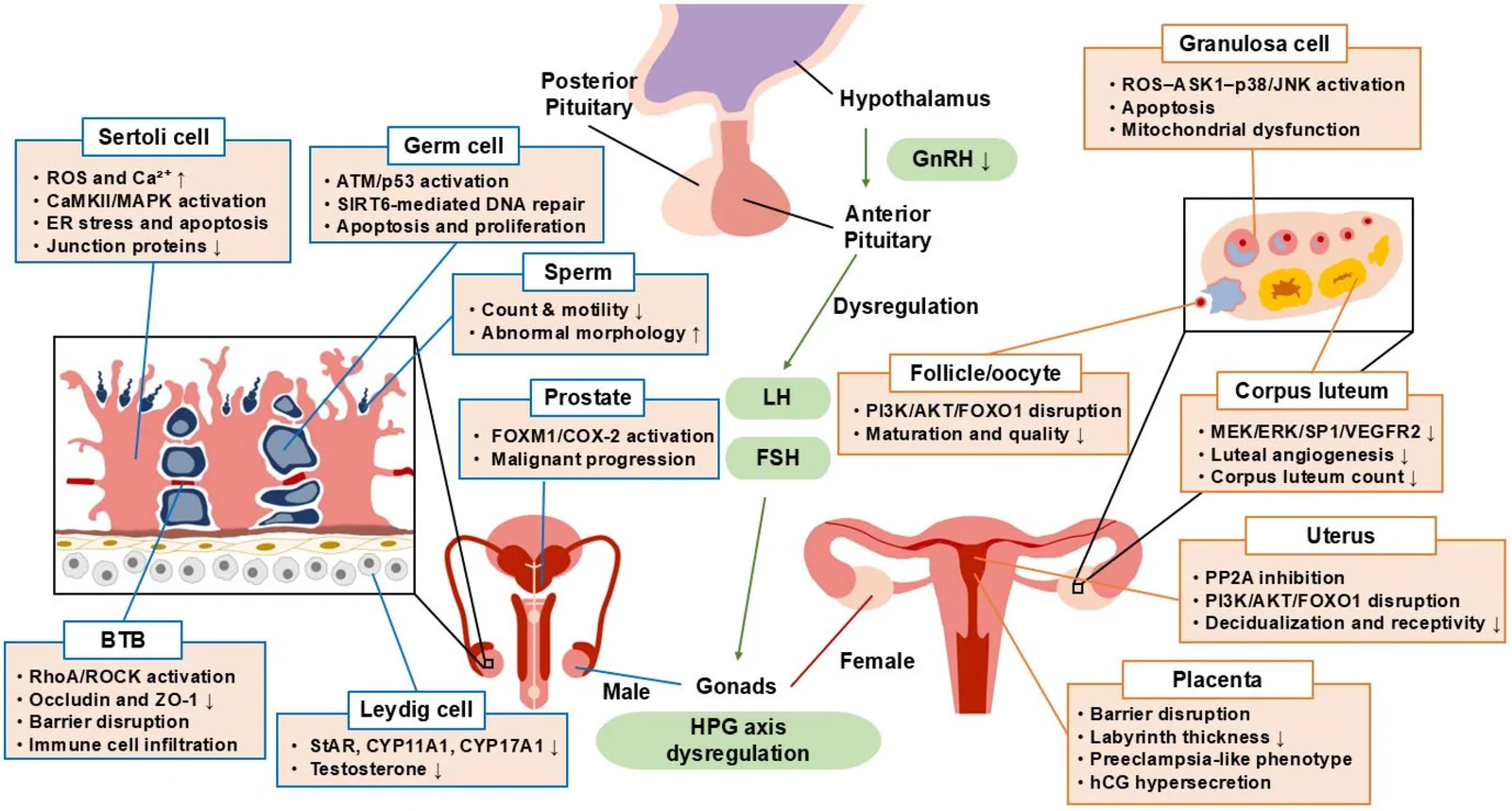
Sex-specific reproductive toxicity of MC-LR in male and female mammals. MC-LR disrupts the HPG axis and induces sex-specific reproductive dysfunction. In males, major effects include Sertoli cell injury, BTB disruption, impaired spermatogenesis, sperm abnormalities, and reduced testosterone. In females, MC-LR impairs granulosa cell function, oocyte quality, uterine receptivity, placental integrity, and steroid hormone balance. Blue elements indicate male-specific effects, orange elements indicate female-specific effects, and green elements indicate shared components.

Testosterone governs key aspects of male reproductive health, including organ development, spermatogenesis, and sexual function, making its signaling pathway a vulnerable target for interference by diverse environmental contaminants. MC-LR acts as an endocrine disruptor by targeting Leydig cells, leading to a significant reduction in serum testosterone levels [53]. In mice, repeated intraperitoneal exposure to MC-LR (20 μg/kg/day for 5 weeks) markedly reduced both serum and intratesticular testosterone levels [39]. Similarly, chronic exposure to MC-LR via drinking water (1–30 μg/L for 6 months) significantly reduced circulating testosterone levels in male mice [40]. The combination of reduced testosterone and elevated LH and FSH is consistent with impaired testicular steroidogenesis and compensatory pituitary activation. However, reduced GnRH and reported hypothalamic and pituitary injury indicate that central HPG axis disruption also contributes to the hormonal imbalance. Consistent with this feedback dysregulation, acute exposure of male mice to MC-LR via intraperitoneal injection causes complex, dose- and time-dependent alterations in reproductive hormone levels [75]. While shorter durations (1–4 days) can initially increase serum LH and testosterone, prolonged exposure (2 weeks) leads to a significant decrease in both hormones. Serum FSH levels follow a similar pattern, with an initial increase followed by a decline at higher doses (30 µg/kg/day). Together, these findings demonstrate that MC-LR disrupts male reproductive hormone homeostasis through coordinated dysregulation of the HPG axis at both central and peripheral levels (Figure 1).

## 3. Female Reproductive Toxicity of MC-LR

### 3.1. Ovarian Toxicity

In parallel with its well-documented effects on the male reproductive system, increasing evidence indicates that MC-LR also exerts profound toxicity on female reproductive organs (Table 2). Accumulating evidence demonstrates that MC-LR can directly affect mammalian ovaries, inducing ovarian atrophy, follicular atresia, steroidogenic imbalance, and inflammatory and stress-related responses that ultimately impair female fertility [76]. In mouse ovarian granulosa KK-1 cells, intracellular accumulation of MC-LR was accompanied by a greater increase in the mRNA expression of Oatp2b1 and Oatp5a1, suggesting that these transporters may contribute to MC-LR uptake into ovarian cells [77]. In female mice, intraperitoneal exposure to 20 µg/kg MC-LR for 4 weeks caused a significant reduction in the gonadosomatic index and a specific decline in the number of primordial follicles, indicating a depletion of the ovarian reserve [78]. Intraperitoneal exposure to MC-LR (40 μg/kg/day for 2 weeks) also induced marked ovarian injury, characterized by follicular atresia, ovarian inflammation, nuclear fragmentation, and a significant reduction in the gonadosomatic index [77,79]. In adolescent female mice, intraperitoneal exposure to MC-LR (10 or 20 μg/kg/day for 2 weeks) resulted in reduced ovarian weight, disrupted follicular development, and a marked decrease in primordial, primary, and secondary follicles, indicating impaired ovarian reserve and folliculogenesis [80]. Moreover, oral exposure to 1, 10, and 40 µg/L MC-LR for 6 months induced pathological changes in the ovary, characterized by a significant reduction in the number of healthy growing follicles and an increase in atretic follicles [81]. Notably, a significant increase in atretic follicles was observed at, a concentration comparable to the WHO guideline value for MC-LR in drinking water [82]. Beyond structural ovarian injury, intraperitoneal MC-LR exposure (7.5–45 μg/kg/day for 2 weeks) leads to marked alterations in ovarian steroidogenic activity, accompanied by abnormal granulosa cell differentiation and disrupted follicular homeostasis [83].

These alterations highlight granulosa cells as a critical cellular mediator of MC-LR-induced ovarian dysfunction. Granulosa cells play a central role in female reproduction by coordinating steroid hormone synthesis that primes the reproductive tract for fertilization and supports the early establishment of pregnancy [91]. Beyond their role in steroidogenesis, granulosa cells actively govern follicular fate decisions, including survival, differentiation, and atresia, thereby directly shaping ovarian reserve dynamics across the reproductive lifespan. Accordingly, heightened susceptibility of granulosa cells to MC-LR represents a critical cellular mechanism underlying follicular depletion, impaired folliculogenesis, and subsequent subfertility observed in exposed females. In recent years, primary-derived granulosa cells have emerged as a preferred in vitro model for ovarian toxicology, as they retain key signaling pathways, steroidogenic capacity, and stress-response mechanisms that closely mirror in vivo ovarian physiology. Using this model system, exposure of ovarian granulosa cells to MC-LR (0.01–1 μM for 24 h) was shown to induce pronounced mitochondrial ultrastructural damage, loss of mitochondrial membrane potential, and increased apoptotic cell death [84]. The lower concentrations of 0.01 and 0.1 μM correspond to approximately 10 and 100 μg/L and overlap with levels reported during active or intense bloom events. The highest concentration of 1 μM represents a high-exposure condition, and nominal culture concentrations cannot be directly equated with ovarian tissue exposure in vivo. Although evidence on female reproductive toxicity has expanded, the male evidence base synthesized in this review includes approximately twice as many distinct mammalian studies. Female evidence also remains concentrated in ovarian and granulosa-cell models, with few chronic in vivo or human studies. This imbalance represents an important knowledge gap.

### 3.2. Disruption of Oocyte Maturation and Ovulation

Building on ovarian structural and steroidogenic disturbances described above, MC-LR exposure also disrupts key functional checkpoints governing oocyte maturation and ovulation. Exposure to 20 µg/kg MC-LR for 4 weeks disrupted the estrous cycle in mice, characterized by significantly shortened proestrus and estrus phases and a prolonged diestrus phase, which may hinder successful ovulation and mating [78]. Notably, long-term oral exposure of female mice to MC-LR (10 μg/kg/day for 6 weeks) did not alter folliculogenesis or estrous cyclicity but resulted in nearly a 50% reduction in the number of corpora lutea, indicating impaired ovulation rather than follicle depletion [85]. Consistent with these findings, chronic intake of 40 µg/L MC-LR for 6 months resulted in female subfertility, evidenced by a prolonged diestrus phase in the estrous cycle and a significantly decreased number of pups per litter [81].

At the gamete level, MC-LR directly deteriorates oocyte competence. Exposure of porcine oocytes to MC-LR during in vitro maturation significantly impaired oocyte nuclear maturation, reduced polar body extrusion, and compromised subsequent embryonic developmental competence [92]. At higher concentrations, treatment with 20 to 60 µM MC-LR for 44 h led to cytoskeletal disruption, characterized by aberrant spindle morphologies and misaligned chromosomes in porcine oocytes [93]. These concentrations greatly exceed environmentally reported levels and were used to investigate cellular mechanisms of oocyte injury. Their relevance to typical environmental exposure is therefore limited. Together, these observations suggest that MC-LR disrupts female fertility through coordinated interference with endocrine regulation, ovulatory processes, and intrinsic oocyte quality. Such impairment at the level of oocyte maturation and ovulation may propagate to later stages of reproduction, affecting implantation and placental development.

### 3.3. Disruption of Pregnancy Maintenance

MC-LR exposure further reduces reproductive success at post-fertilization stages. Maternal exposure to MC-LR during the critical period of organogenesis has been shown to induce significant toxicity in pregnant mice. At the concentration of 12 µg/kg, MC-LR induced the reduced maternal weight gain, pregnancy termination, and even mortality, indicating a threat to pregnancy maintenance [94]. Consistent with these observations, intraperitoneal exposure to MC-LR (15 μg/kg/day) during early pregnancy significantly reduced the number of implantation sites and embryo size in pregnant mice, indicating impaired embryo implantation and early pregnancy maintenance [87]. These findings suggest that MC-LR interferes not only with maternal physiology but also with the establishment of a supportive uterine environment required for successful implantation. Mechanistically, MC-LR impairs luteal angiogenesis by downregulating VEGFR2 expression in vascular endothelial cells, thereby disrupting vascular network formation essential for corpus luteum function. Given that the corpus luteum serves as the primary endocrine source of progesterone during early pregnancy, such vascular insufficiency directly weakens hormonal support necessary for embryo survival. Collectively, these data indicate that MC-LR-induced endocrine and vascular dysfunction at the maternal–fetal interface creates a permissive environment for implantation failure and early pregnancy loss, setting the stage for subsequent placental abnormalities.

Beyond implantation and luteal function, MC-LR may also compromise pregnancy maintenance through placental injury. The placenta serves as a dynamic interface between maternal and fetal circulations, functioning not only as a physical barrier but also as a critical regulator of immune tolerance and endocrine signaling throughout pregnancy. In rodent models, MC-LR exposure has been associated with disruption of placental structure and barrier-related architecture [95]. Following systemic exposure, MC-LR is preferentially distributed to the placenta during pregnancy, where it accumulates within the highly vascularized and rapidly expanding placental circulation [88]. Histological and ultrastructural analyses further revealed that MC-LR exposure disrupted placental labyrinth architecture, characterized by reduced maternal-fetal vascular space, endoplasmic reticulum (ER) dilation, particularly at 20 mg/kg [95]. Such alterations may increase fetal glucocorticoid exposure and limit nutrient supply, thereby undermining pregnancy maintenance and fetal growth. Dysregulation of placental endocrine and vascular function is a recognized pathogenic feature of hypertensive pregnancy disorders. Preeclampsia is a major cause of maternal morbidity and mortality and is closely linked to fetal growth restriction, with accumulating evidence indicating that it also predisposes offspring to an increased risk of cardiovascular disease [96]. Defective placental invasion and spiral artery remodeling are central pathological features of preeclampsia, resulting in placental hypoxia and widespread maternal endothelial injury that underlie both vascular and metabolic complications during pregnancy. Prenatal exposure to MC-LR in pregnant mice (0.04 or 0.2 mg/kg/day) induced preeclampsia-like phenotypes, including maternal hypertension, proteinuria, reduced placental labyrinth thickness, and impaired uteroplacental vascularization [89]. These findings indicate the sensitivity of the maternal-fetal interface to MC-LR during pregnancy. However, susceptibility to MC-LR appears to vary across placental cell types and experimental contexts. Human placental villous trophoblasts appeared relatively resistant to MC-LR in vitro, maintaining cell viability and morphological differentiation into syncytiotrophoblasts after exposure to concentrations up to 20 µM for 48 h [97]. This finding suggests that placental susceptibility may differ across species and experimental systems. It also limits direct extrapolation of rodent placental lesions to human trophoblast cytotoxicity. However, preserved trophoblast viability does not exclude human placental risk because vascular and endocrine dysfunction may emerge at the tissue level. Human villous trophoblasts express OATP1B3 in cytotrophoblasts and syncytiotrophoblasts, suggesting a route for MC-LR uptake [97]. Although cell viability and differentiation were largely preserved in vitro, prolonged exposure altered hCG secretion. Placental toxicity may therefore occur through functional endocrine and vascular disturbances without overt trophoblast death, consistent with the placental lesions observed in vivo [89,95]. However, transplacental transfer efficiency and fetal tissue distribution remain unclear because MC-LR has not been measured simultaneously in maternal blood, placenta, fetal circulation, and multiple fetal organs. In parallel, successful endometrial receptivity is critically dependent on the precise timing and duration of progesterone signaling following sufficient estrogen priming. Consistent with this endocrine vulnerability, intraperitoneal exposure to MC-LR at doses of 15 and 30 µg/kg/day from GD1 to GD8 in mice has been shown to significantly reduce uterine weight, the number of implantation sites, and the weight of those sites by impairing the process of endometrial decidualization [86]. Collectively, these findings indicate that MC-LR disrupts pregnancy maintenance through coordinated disruption of placental vascular function and uterine endocrine responsiveness.

### 3.4. Disruption of Female Reproductive Hormone Homeostasis

Reproductive toxicants can impair ovarian function through multiple pathways, either by exerting direct cytotoxic or functional effects on ovarian tissue or by indirectly disrupting hormonal signaling along the hypothalamic-pituitary axis. In the context of MC-LR exposure, growing evidence suggests that both mechanisms may operate in a context- and dose-dependent manner. Experimental studies in mammals indicate that MC-LR exposure interferes with ovarian steroidogenesis, resulting in disrupted progesterone and estrogen homeostasis and abnormal estrous cyclicity [71]. At the systemic level, GnRH is pivotal in the neurohormonal regulation of reproduction, primarily by stimulating the pituitary gland to release FSH and LH. Long-term exposure to MC-LR (40 µg/L for 6 months) led to significant reductions in circulating FSH, LH, estradiol, and progesterone levels, indicative of suppression of the HPG axis [81]. However, not all hormonal disturbances appear to originate from central neuroendocrine dysfunction. The variability observed in experimental outcomes using in vitro granulosa cell cultures likely reflects the significant inter-individual differences in physiological parameters, such as plasma progesterone concentrations, found among naturally cycling women in vivo [98]. Supporting a primary ovarian origin, repeated MC-LR exposure (5 or 20 µg/kg for 4 weeks) significantly reduced serum progesterone levels without altering FSH, LH, or estradiol concentrations, suggesting direct ovarian dysfunction rather than hypothalamic–pituitary impairment [78]. Additional studies further highlight the complexity of MC-LR–induced endocrine disruption. Following a single injection of environmentally relevant doses (5–40 µg/kg MC-LR), female rats exhibit a profound hormonal imbalance within 15 days, including significantly reduced serum estradiol levels alongside elevated levels of progesterone and anti-Müllerian hormone [90]. Similarly, both in vitro exposure of mouse granulosa cells and in vivo exposure of female mice resulted in a significant increase in progesterone production, indicating that MC-LR can directly disturb ovarian steroid hormone balance [83]. Importantly, endocrine disruption by MC-LR is not confined to the ovary. MC-LR exposure induced a dose-dependent increase in the secretion of human chorionic gonadotropin (hCG), a critical pregnancy hormone, in trophoblasts exposed to 5–10 µM MC-LR for 72 h, indicating a functional disruption despite the lack of morphological damage [97]. Together, these findings suggest that MC-LR disrupts female reproductive endocrinology through coordinated but heterogeneous effects on central regulation, ovarian steroidogenesis, and placental hormone signaling.

## 4. Developmental Toxicity of MC-LR

Beyond its immediate effects on adult reproductive organs and endocrine regulation, accumulating evidence suggests that MC-LR exposure can exert lasting consequences on early development and offspring health (Table 3). Such effects may arise when toxic insults occur during sensitive windows of gametogenesis, fertilization, and early embryogenesis, thereby propagating adverse outcomes across developmental stages. Given that oocyte quality is a key determinant of early embryonic development, MC-LR-induced oxidative damage during oocyte maturation may represent an early initiating event linking maternal exposure to later developmental toxicity in offspring [92]. This early germline vulnerability provides a plausible mechanistic foundation for the widespread developmental abnormalities observed following gestational MC-LR exposure. Exposure to MC-LR during gestation is consistently associated with severe developmental deficits, including retarded fetal growth, gross structural anomalies, and increased rates of embryonic death. Gestational exposure to MCs (specifically a mix of 98% MC-LR and 2% MC-YR) causes dose-dependent embryotoxicity, characterized by a significant increase in embryo resorption and death at higher concentrations [94]. Importantly, developmental toxicity is not restricted to embryonic lethality, as surviving offspring frequently display persistent growth and structural abnormalities. Surviving fetuses may exhibit significant growth restriction, with notable reductions in body weight, body length, and tail length. Cardiac development was also affected, as prenatal exposure to MC-LR (0.04 or 0.2 mg/kg/day during gestation) induced pronounced cardiac malformations in mouse embryos, including ventricular dilation and myocardial thinning [88]. The lowest prenatal dose of 40 μg/kg/day overlaps with doses reported to induce testicular toxicity in adult rodents. Thus, the available evidence suggests broadly similar dose ranges, although direct comparison is limited by differences in exposure route, duration, life stage, and evaluated endpoints. Beyond gross morphological defects, increasing evidence indicates that MC-LR disrupts organ-specific developmental programs during the perinatal period. Perinatal exposure to 1, 10, and 50 µg/L MC-LR from gestational day (GD) 12 to postnatal day (PND) 21 significantly impaired prostate development in male mice, resulting in reduced ventral prostate weight and inhibited ductal branching morphogenesis [99]. Similarly, maternal exposure to MC-LR at a concentration of 10 µg/L via drinking water from GD 12 to PND 21 has been shown to induce prostate hyperplasia in male offspring, highlighting the toxin’s capability to disrupt the development of male accessory sex glands [100]. Mechanistically, exposure during this critical period of prostate morphogenesis may alter both endocrine and proliferative programs. MC-LR disrupted aromatase-dependent androgen-estrogen balance and AR/ERα signaling, while downregulation of PIWI proteins and piRNA-DQ722010 activated the PI3K/AKT pathway, promoting abnormal epithelial proliferation. These changes provide a plausible link between perinatal exposure and persistent prostate abnormalities in the offspring. These effects are not confined to the reproductive system, but extend to multiple developing organs with long-term functional consequences. Perinatal exposure to 10 µg/kg MC-LR daily from GD 8 to PND 15 resulted in significant toxin accumulation in the brains of rat offspring, leading to ultrastructural damage and neurodevelopmental impairments [101]. Maternal exposure to 10 µg/kg MC-LR daily from GD 8 to PND 15 also resulted in significant liver impairment in rat offspring, characterized by widened cell junctions and increased lipid droplets in hepatocytes [102]. Such widespread developmental perturbations provide a critical framework for understanding how MC-LR exposure may exert effects that extend beyond the directly exposed generation.

**Figure 2:**
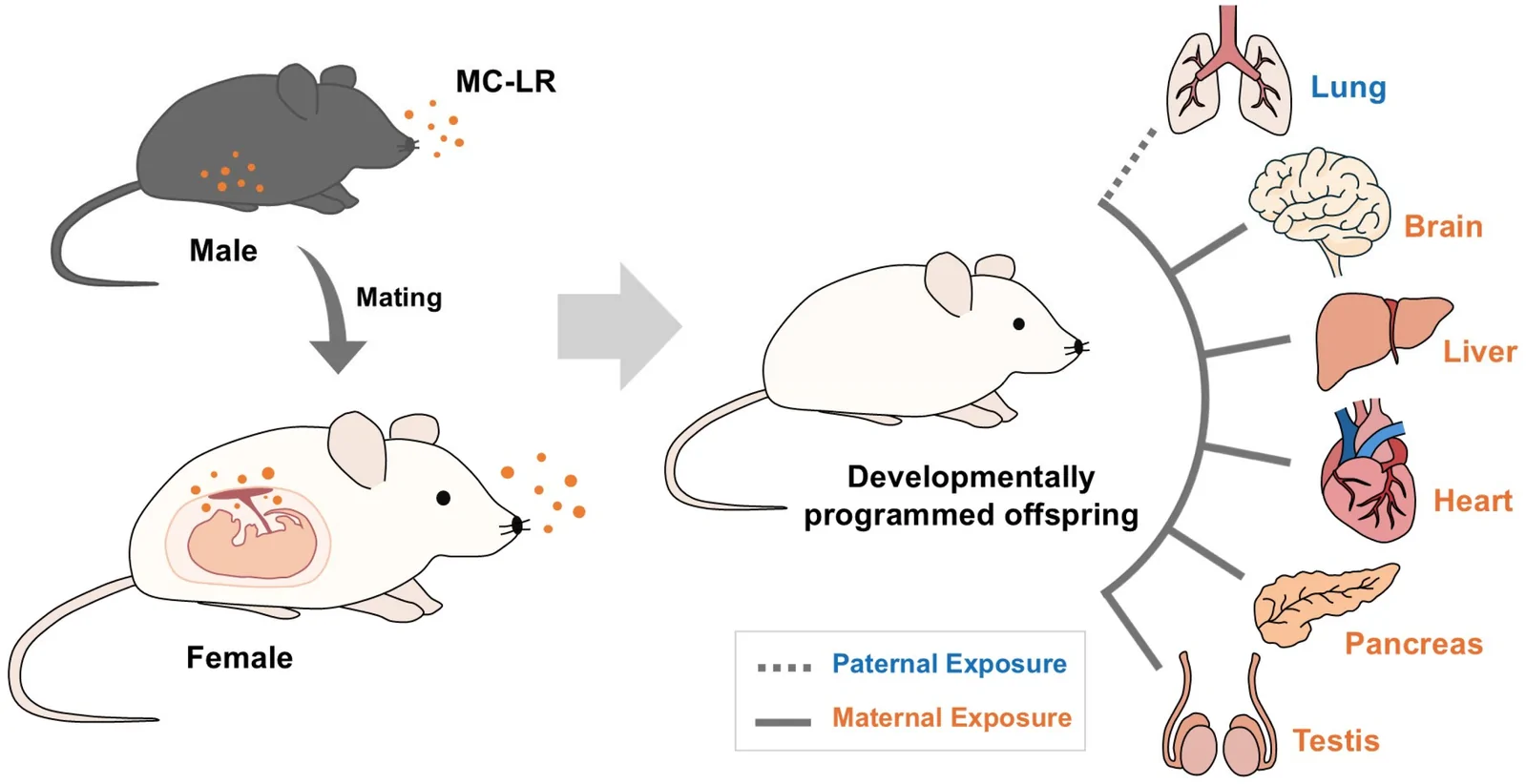
Developmental programming and multi-organ consequences in offspring following parental exposure to MC-LR. Both paternal and maternal exposure contribute to long-term developmental and organ-specific impairments in offspring, with paternal exposure affecting the lung and maternal exposure affecting the brain, liver, heart, pancreas, and reproductive organs. Dotted and solid lines indicate paternal and maternal exposure, respectively.

Importantly, the developmental effects are not limited to immediate structural or functional abnormalities during gestation, but may persist beyond birth and influence developmental trajectories at later life stages. Parental exposure to MC-LR not only impairs embryonic development but also induces long-term pathological lesions in various organs of the offspring across multiple animal models. Paternal exposure to MC-LR via drinking water at 10 µg/L prior to conception has been linked to lung-related pathologies and altered ontogenetic development in offspring [104]. Notably, cardiac abnormalities induced by prenatal MC-LR exposure persisted into postnatal life, as offspring exhibited reduced cardiac output, impaired systolic function, and increased myocardial fibrosis at 4 weeks of age [88]. Maternal exposure to 10 µg/L MC-LR via drinking water throughout the gestational and lactational periods significantly reduced testis weight and impaired sperm concentration and motility in the adult male offspring [47]. The prostate was also affected, as maternal exposure to MC-LR during the critical window of pregnancy and lactation induces long-lasting developmental defects in the prostate of male offspring, which may predispose them to reproductive dysfunction in adulthood [99]. Male offspring prenatally exposed to MC-LR (30 μg/L) further exhibited impaired glucose tolerance and paradoxically elevated fasting serum insulin levels at 4 weeks of age, indicating persistent dysregulation of glucose-insulin homeostasis extending beyond the neonatal period [103]. Developmental programming refers to persistent alterations in organ development and later-life disease susceptibility following an insult during a critical developmental window [105]. Such insults may redirect developmental trajectories through lasting changes in epigenetic regulation, including DNA methylation, histone modifications, and non-coding RNAs, together with altered endocrine signaling. Collectively, these findings indicate that MC-LR exposure during critical developmental windows can influence developmental trajectories and contribute to persistent, multi-organ dysfunction extending into postnatal life and adulthood (Figure 2). Such sustained developmental perturbations suggest that MC-LR exposure may increase susceptibility to metabolic, cardiovascular, and reproductive disorders later in life. However, the relative severity of paternal and maternal exposure cannot be directly compared because paternal evidence remains limited and the available studies differ in exposure design and organ-specific endpoints. The broader range of outcomes reported after maternal exposure therefore reflects the current evidence base and does not necessarily indicate greater toxic potency. Notably, these studies differ substantially in species, dose, exposure route, and evaluated endpoints. Comparable adolescent-exposure models are also lacking. The available evidence therefore does not permit a quantitative ranking of developmental windows or identification of a definitive highest-susceptibility stage for each organ.

## 5. Mechanisms of MC-LR-Induced Reproductive and Developmental Toxicity

Cellular responses to toxicants are inherently complex, varying with exposure concentration, duration, and cellular context, triggering a cascade of downstream events that affect the cytoskeleton, cell cycle, and apoptosis. In this regard, MC-induced toxicity is best understood as a multifaceted process involving the integration of several interconnected molecular pathways [106]. MC-induced toxicity is conceptualized as a phosphatase-centered process, in which inhibition of serine/threonine protein phosphatases serves as a primary initiating event. By inhibiting the catalytic activity of PP1 and PP2A, MCs induce the widespread hyperphosphorylation of numerous protein substrates. This dysregulates vital cellular pathways, including the apoptotic cascade, by altering the phosphorylation status of its key regulatory proteins [107]. In ovarian cells, MC-LR-mediated inhibition of PP2A contributes to sustained phosphorylation of MAP3K5, thereby amplifying ER stress signaling [84]. In granulosa cells, MC-LR selectively inhibits PP1 activity, disrupting PP1-mediated PI3K/AKT/FOXO1 signaling that is essential for FSH-dependent follicle maturation [85]. In the uterus, MC-LR-induced decidualization failure is initiated by the inhibition of PP2A activity. This inhibition serves as a critical upstream event that triggers the disruption of endometrial stromal cell differentiation [86]. Such phosphatase-dependent toxicity presupposes that sufficient intracellular concentrations of MC-LR are achieved, implicating cellular uptake mechanisms as a key determinant of tissue-specific vulnerability.

**Figure 3:**
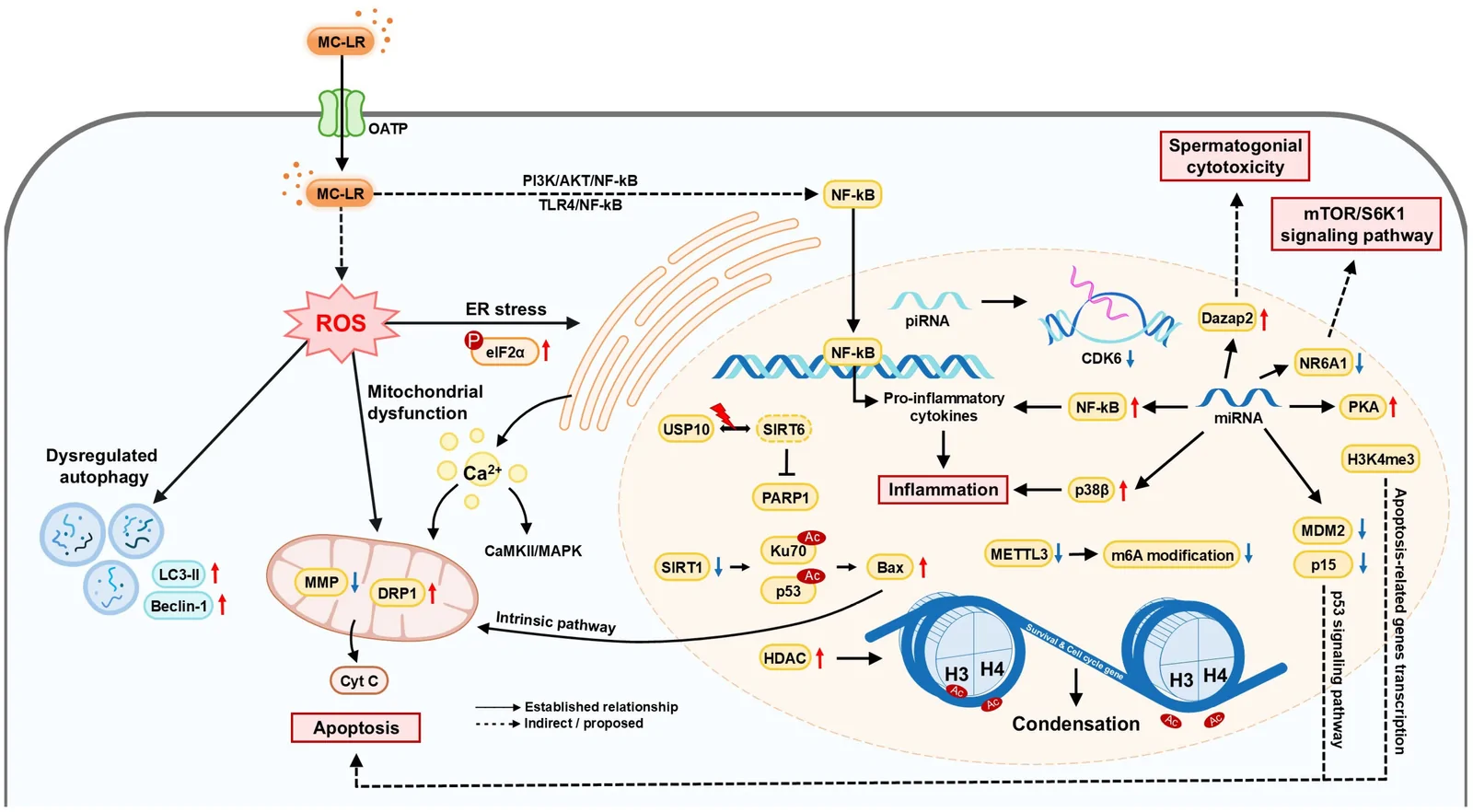
Integrated molecular mechanisms underlying MC-LR-induced reproductive and developmental toxicity. MC-LR uptake triggers ROS generation, ER stress, mitochondrial dysfunction, Ca^2+^ dyshomeostasis, and dysregulated autophagy. These events converge on inflammatory signaling, DNA damage and repair failure, non-coding RNA dysregulation, epigenetic remodeling, and apoptotic pathways, ultimately leading to germ cell cytotoxicity and impaired spermatogenesis. Solid arrows indicate established pathway relationships, whereas dashed arrows indicate indirect or proposed links. Red upward and blue downward arrows indicate increased and decreased expression or activity, respectively. Arrow direction represents a conceptual progression from MC-LR uptake to secondary stress responses and downstream cellular outcomes, not a strict temporal sequence.

The cellular uptake of MC-LR is mediated predominantly by specific transporters, OATPs, whose expression levels are altered following MC-LR exposure [65]. While the liver is the primary target due to high transporter expression, recent studies have identified the presence of OATPs in reproductive tissues, providing a molecular basis for the reproductive toxicity of MC-LR. Rat spermatogonia express Oatp1a5, Oatp3a1, Oatp6b1, Oatp6c1, and Oatp6d1, with Oatp3a1 showing the most prominent response to MC-LR [65]. In mouse ovarian cells, Oatp2b1 and Oatp5a1 show greater MC-LR-induced increases than other detected Oatps, whereas human villous trophoblasts express OATP1B3 [77,97]. Direct comparison of transporter abundance among these tissues is not currently possible because the available studies differ in species, cell type, and analytical method. Once internalized, MC-LR can engage diverse stress-adaptive and regulatory pathways in a context-dependent manner, ultimately influencing cellular survival, genomic integrity, and hormonal signaling. Although previous systemic reviews have provided a macroscopic view of MC-LR’s toxicokinetics and general organ toxicity, there is still a significant need for a more granular analysis focusing on the unique vulnerability of the reproductive and developmental axis [29]. The following sections therefore examine these interconnected molecular events in detail that collectively drive MC-LR-induced reproductive and developmental toxicity (Figure 3).

### 5.1. Cellular Stress Response

#### 5.1.1. Oxidative Stress

Cellular stress responses serve as early adaptive mechanisms to toxic insults. However, sustained or excessive activation of these pathways can overwhelm protective capacity and precipitate irreversible cellular damage. Oxidative stress is among the most frequently reported responses to MC-LR exposure, arising from increased generation of reactive oxygen species (ROS) and depletion of glutathione (GSH), and contributing to cytotoxicity, apoptosis, and DNA damage across cell types [28,29]. Oxidative stress is a major contributor to MC-LR-induced testicular injury, as supported by rapid increases in lipid peroxidation and ROS and by antioxidant interventions that attenuate mitochondrial injury and apoptosis [56,108]. MC-LR exposure further disrupts homeostasis in the testis by causing significant, time-dependent alterations to the transcription of key antioxidant enzyme genes [109]. This includes the notable upregulation of genes for manganese superoxide dismutase (Mn-SOD) and glutathione peroxidase (GPx), indicating a direct toxic effect on the genetic regulation within testicular cells. These results indicate that exposure to MC-LR triggers a compensatory defense mechanism involving the active induction of the cellular antioxidant enzyme system. Consistent with this notion, cell-type-specific studies reveal that antioxidant defenses are differentially dysregulated across testicular cell populations. In vitro experiments on Leydig cells showed that MC-LR exposure increased the production of ROS, enhanced lipid peroxidation, and depleted the activity of the critical antioxidant enzyme superoxide dismutase (SOD) [49]. Similarly, MC-LR induces significant oxidative stress in Sertoli cells, characterized by increased production of ROS and lipid peroxidation, coupled with decreased SOD activity, with effects becoming significant at concentrations as low as 5 nM [110]. Together, these findings suggest that although antioxidant responses are transcriptionally induced, sustained MC-LR exposure overwhelms cellular redox buffering capacity, leading to persistent oxidative injury within the testicular microenvironment. The critical role of oxidative stress in MC-LR toxicity was further substantiated by the observation that pretreatment with N-acetyl-L-cysteine (NAC), a potent ROS scavenger, effectively attenuated intracellular oxidative injury and subsequent cell death [56]. Comparable oxidative stress responses have also been documented in female reproductive cells, indicating that redox imbalance represents a conserved mechanism of MC-LR toxicity across sexes. In ovarian granulosa cells, MC-LR induced excessive ROS production and inhibited antioxidant enzymes (SOD, CAT, GPx), leading to oxidative stress-mediated apoptosis [81]. MC-LR also induces oxidative stress in oocytes by elevating intracellular ROS levels, which subsequently triggers early apoptosis evidenced by phosphatidylserine externalization and increased caspase-3 activity [93]. These findings support a causal role for ROS in downstream cellular injury. However, they do not establish whether ROS generation occurs downstream of PP1/PP2A inhibition. The temporal relationship between these mechanisms remains unresolved, and they may interact in a context-dependent manner.

#### 5.1.2. Mitochondrial Dysfunction

Due to their central role in cellular energy metabolism, redox homeostasis, and apoptosis signaling, mitochondria represent critical and vulnerable targets for a wide array of toxicants [111]. MC-LR also triggered the mitochondrial apoptotic pathway in Sertoli cells through excessive ROS generation, which led to the collapse of mitochondrial membrane potential and the subsequent activation of caspase-3 and caspase-9 [56]. MC-LR further triggers the p53-dependent apoptotic pathway, which subsequently promotes the opening of the mitochondrial permeability transition pore and leads to the release of cytochrome c from the mitochondria into the cytoplasm [112]. MC-LR exposure also results in mitochondrial swelling and mitochondrial DNA damage in testis. This mitochondrial stress is further evidenced by the significant compensatory upregulation of oxidative phosphorylation genes [52]. MC-LR disrupts mitochondrial dynamics by promoting excessive mitochondrial fission through up-regulation of dynamin-related protein 1 (DRP1), thereby shifting the balance toward a fragmented mitochondrial network associated with cellular dysfunction [84].

#### 5.1.3. Endoplasmic Reticulum (ER) Stress

Oxidative stress and subsequent Ca^2+^ dyshomeostasis are major triggers of ER stress, a condition that disrupts cellular proteostasis and promotes apoptosis when sustained. Proteomic analysis revealed that MC-LR-induced developmental neurotoxicity is mediated by ER stress, as evidenced by the alteration of proteins involved in protein folding and degradation [101]. In Sertoli cells, MC-LR disrupts intracellular Ca^2+^ homeostasis, leading to the phosphorylation of calmodulin-dependent protein kinase II (CaMKII) and the subsequent activation of MAPK signaling pathways (p38, JNK, and ERK), which collectively trigger mitochondrial dysfunction and apoptosis [36]. In Leydig cells, MC-LR induces excessive ROS generation, leading to activation of the integrated stress response via GCN2-dependent phosphorylation of eIF2α, a pathway known to suppress global protein translation under cellular stress conditions [39]. These findings indicate that MC-LR-induced oxidative stress converges on Ca^2+^ dysregulation and ER stress signaling, culminating in translational arrest, mitochondrial dysfunction, and apoptotic cell death.

#### 5.1.4. Dysregulated Autophagy

Autophagy is an evolutionarily conserved catabolic process that is activated in response to a wide range of cellular stressors, including oxidative stress, ER stress, and DNA damage. MC-LR triggers autophagy and causes a dysfunctional accumulation of autophagosomes in some cell types. Autophagosome accumulation subsequently promotes apoptosis, characterized by a significant decrease in anti-apoptotic Bcl-2 and the activation of cytochrome C and caspase-3 in Sertoli cells [58]. Oxidative stress-mediated DNA damage activates the ATM/CHK2/p53 signaling cascade, which in turn promotes autophagy through up-regulation of core autophagy-related proteins such as Beclin-1, ATG5, ATG12, and LC3-II in male germ cells [34]. MC-LR also induces excessive autophagy in ovarian granulosa cells, as evidenced by increased LC3-II and Beclin-1 expression, reduced P62 levels, and enhanced autophagosome-autolysosome formation [113]. Thus, excessive or dysregulated autophagy emerges as a key downstream consequence of MC-LR-induced cellular stress across both male and female reproductive cells.

#### 5.1.5. Inflammatory Signaling

Mounting evidence indicates that MC-LR–induced cellular stress is accompanied by the activation of inflammatory signaling pathways within reproductive tissues. MC-LR exerts immunomodulatory effects in testicular cells by activating the PI3K/Akt/NF-κB signaling pathway, which upregulates the expression of pro-inflammatory cytokines [114]. In Sertoli cells, MC-LR initiates an inflammatory cascade by activating the TLR4/NF-κB signaling pathway, which serves as a primary sensor for the toxin [59,115]. In prostate epithelial cells, MC-LR triggers an inflammatory response in the prostate by activating the NF-κB signaling pathway, which induces the overexpression of pro-inflammatory cytokines such as TNF-α and IL-6 [70]. Ovarian inflammation is increasingly recognized as a critical factor that damages oocyte competence and ovulatory capacity, thereby promoting infertility through disruption of the follicular microenvironment. MC-LR induces mitochondrial dysfunction in ovarian granulosa cells, leading to the cytosolic leakage of mitochondrial DNA, a key trigger of cellular stress-associated inflammatory responses [116]. Collectively, these findings position inflammation as an integral component of MC-LR-induced cellular stress, leading to reproductive tissue injury.

### 5.2. Genotoxicity and Epigenetic Regulation

Cyanobacterial bloom extracts exhibit potent genotoxic activity, inducing significant, dose-dependent DNA damage in testicular cells [117]. Accumulating evidence indicates that MC-LR is a potent genotoxic agent capable of inducing DNA damage across multiple mammalian cell types, including hepatocytes and human peripheral blood lymphocytes [118]. Notably, this genotoxic effect was found to be even more pronounced in testicular cells than in liver cells, highlighting the heightened vulnerability of germ cells to DNA damage. Consistent with this genotoxic response, p53, a critical sensor of cellular stress and DNA damage, is significantly upregulated in the testis following MC exposure [119]. While MC-LR is generally not considered directly mutagenic in standard bacterial assays, it induces secondary genotoxicity in vitro and in vivo, leading to DNA strand breaks, micronuclei formation, and potentially interfering with DNA repair mechanisms [120].

p53 is implicated in MC-LR-induced Sertoli cell apoptosis, showing significantly increased mRNA and protein expression levels [55]. This upregulation likely contributes to the transcriptional activation of Bax and the subsequent initiation of the apoptotic cascade. The intrinsic apoptotic pathway, mediated by mitochondria, commences with the release of cytochrome c, which triggers the assembly of the apoptosome [121]. This complex subsequently activates the initiator caspase-9, leading to the activation of the executioner caspase-3 and culminating in the characteristic fragmentation of nuclear DNA. MC-LR-induced genotoxicity involves the induction of DNA double-strand breaks and the simultaneous failure of DNA repair mechanisms through the proteasomal degradation of SIRT6 [37]. Upon the induction of genomic instability, Poly(ADP-ribose) polymerase 1 (PARP1) acts as a molecular scaffold through its catalytic PARylation activity to initiate the repair cascade [122]. MC-LR triggers the disruption of the SIRT6-USP10 interaction, which in turn inhibits PARP1-mediated PARylation activity required for effective oxidative DNA damage repair. Moreover, MC-LR inhibits the expression of SIRT1, a NAD^+^-dependent deacetylase, leading to the hyperacetylation of p53 and Ku70 in Sertoli-germ cells [108]. The acetylation of Ku70 disrupts its binding with Bax, thereby releasing Bax to translocate to the mitochondria and initiate apoptosis, a process that can be reversed by SIRT1 activators.

Beyond protein-coding genes, MC-LR profoundly reshapes the non-coding RNA landscape in the testis, including microRNAs (miRNAs), PIWI-interacting RNAs (piRNAs), circular RNAs (circRNAs), and long non-coding RNAs (lncRNAs), forming a complex network that modulates spermatogenesis-related gene expression [64]. MiRNAs are small non-coding RNAs that function as critical post-transcriptional regulators by binding to the 3′-untranslated regions (UTRs) of target mRNAs to induce gene silencing. These regulatory mechanisms are particularly vital for spermatogenesis, a process that relies heavily on precise post-transcriptional control to orchestrate spermatogonial proliferation, meiotic division, and the morphological differentiation of spermatids [123]. MC-LR altered the expression profile of miRNAs in Sertoli cells, which in turn downregulated target genes involved in tight junctions, leading to structural impairment [57]. Exposure to MC-LR in male germ cells significantly downregulated miR-96 expression, which in turn upregulated its target gene *Dazap2*, leading to spermatogonial cytotoxicity [66]. MC-LR also significantly downregulated the expression of miR-98-5p and miR-758 in Sertoli cells [50]. The suppression of these miRNAs led to the translational activation of their target gene, *MAPK11*, thereby initiating an inflammatory signaling cascade. In bovine Sertoli cells, miR-149-3p serves as a critical negative regulator that directly targets the 3′ UTR of NF-κB, and its suppression by MC-LR leads to the loss of translational inhibition of pro-inflammatory signaling [124]. MC-LR also exerts post-transcriptional control over the HPG axis by downregulating miR-329-3p, which directly targets the 3′-UTR of the regulatory and catalytic subunits of protein kinase A (PKA) to inhibit GnRH synthesis [73]. Meanwhile, MC-LR upregulated miR-541 in spermatogonial cells. This increase suppressed the expression of MDM2 and p15, thereby activating the p53 signaling pathway and promoting apoptosis [125].

Regulation of noncoding RNAs also plays a key role in the female reproductive toxicity and longer-term reproductive consequences of MC-LR exposure. In ovarian granulosa cells, the toxicity of MC-LR involves a complex epigenetic regulatory network where differentially expressed miRNAs target key genes to modulate cellular growth and DNA damage responses [126]. In placental macrophages, MC-LR-induced upregulation of trophoblast-derived miR-377-3p leads to suppression of the nuclear receptor NR6A1, thereby activating downstream mTOR signaling and its effector S6K1 [88]. Beyond somatic cells, emerging evidence implicates small noncoding RNAs in the transmission of MC-LR-induced reproductive dysfunction across generations. The transgenerational reproductive toxicity of MC-LR is linked to the dysregulation of testicular piRNAs. MC-LR-induced upregulation of piR_003399 suppressed its target cyclin-dependent kinase 6 (CDK6) and downstream Rb phosphorylation, thereby impairing spermatogonial proliferation [47]. In sperm, MC-LR exposure alters the expression profile of piRNAs, which subsequently disrupts the Wnt/β-catenin signaling pathway in the offspring, leading to developmental abnormalities [104]. In the prostate of male offspring, reduced PIWI proteins and piRNA-DQ722010 increased the expression of its validated target Pik3r3, leading to PI3K/AKT activation and epithelial hyperplasia [100]. Collectively, these findings identify noncoding RNAs as key epigenetic mediators through which MC-LR reshapes gene regulatory networks, influencing reproductive function and developmental outcomes beyond the directly exposed tissues.

In addition to RNA-mediated post-transcriptional regulation, MC-LR profoundly alters epigenetic control at the chromatin and RNA modification levels, further reinforcing its capacity to reprogram reproductive cell fate. MC-LR has been shown to profoundly alter epigenetic regulation in testicular cells by enhancing histone deacetylase (HDAC) activity and reducing acetylation levels of histones H3 and H4 [127]. This epigenetic shift represses transcriptional programs essential for germ cell survival and cell-cycle progression, positioning histone acetylation as a key mediator of MC-LR-induced reproductive toxicity. MC-LR also promotes trimethylation of histone H3 at lysine 4 (H3K4me3) in testicular cells, leading to transcriptional activation of multiple apoptosis-related genes [128]. Beyond chromatin remodeling, MC-LR also interferes with post-transcriptional epigenetic regulation. Specifically, MC-LR downregulates the m6A methyltransferase METTL3 in Sertoli cells, leading to reduced m6A modification of *Ldha* mRNA and decreased transcript stability [129]. Together, these alterations highlight epigenetic dysregulation as a convergent mechanism through which MC-LR disrupts gene expression at multiple regulatory layers, ultimately compromising reproductive cell integrity.

### 5.3. Endocrine Disrupting Mechanisms

**Figure 4:**
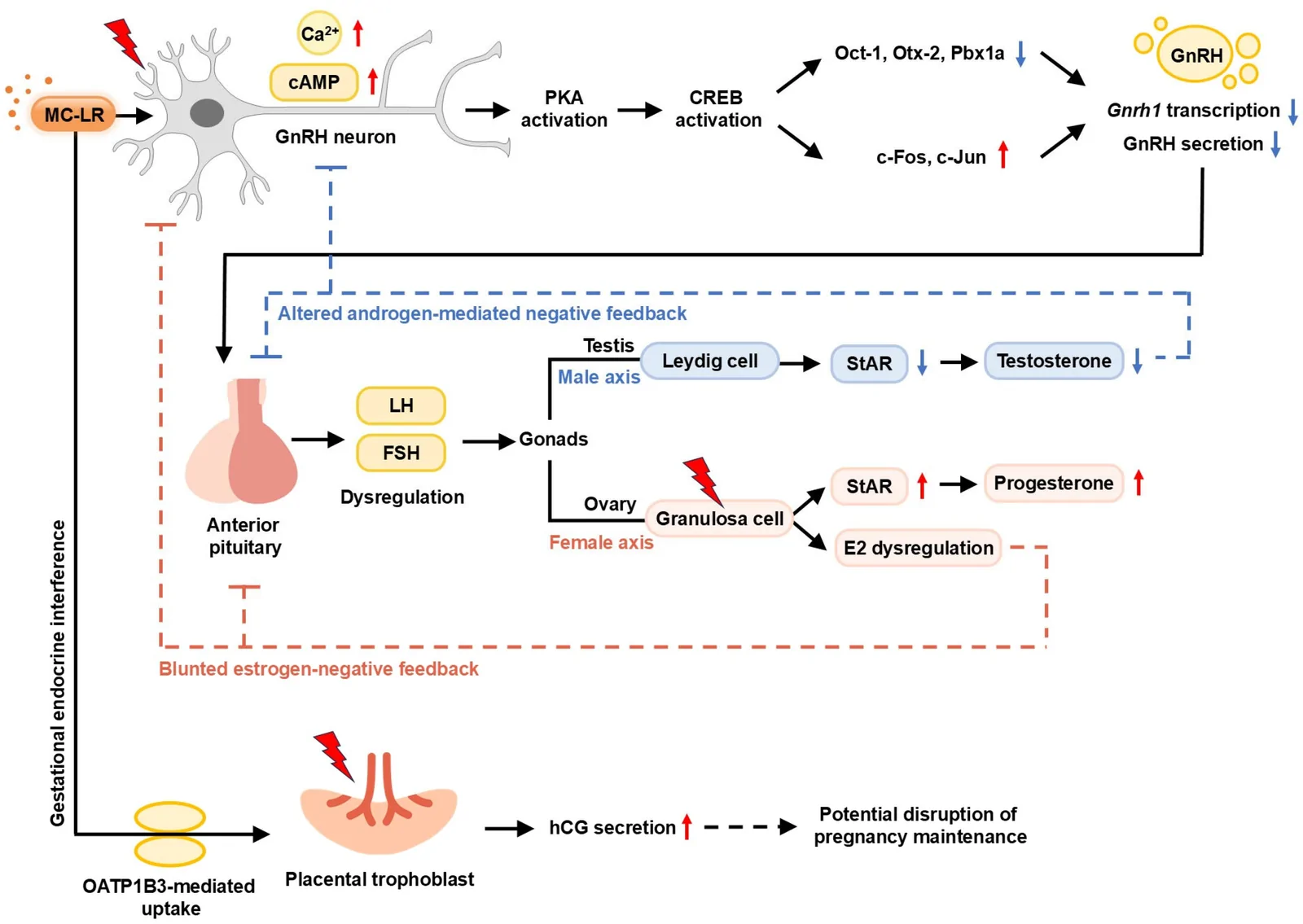
MC-LR-induced disruption of the HPG axis and placental endocrine signaling. In GnRH neurons, MC-LR activates Ca^2+^/cAMP–PKA/CREB signaling, suppressing *Gnrh1* transcription and GnRH secretion. In males, testicular StAR expression and testosterone production are reduced, whereas in females, granulosa-cell StAR expression and progesterone production are increased alongside estradiol dysregulation. Proposed OATP1B3-mediated uptake by placental trophoblasts is associated with increased hCG secretion. Red lightning symbols indicate cellular sites of functional alteration, not direct molecular binding. Blue and orange dashed lines indicate male and female negative feedback, respectively, whereas the black dashed arrow indicates a proposed downstream consequence. Red upward and blue downward arrows indicate increases and decreases, respectively.

Given that the endocrine system encompasses a vast network of organs and cells involved in hormonal signaling, the impact of endocrine-disrupting chemicals (EDCs) is rarely localized. This broad functional scope explains why MC-LR exposure often leads to multi-organ dysfunction by interfering with various hormonal feedback loops (Figure 4). Experimental evidence from sub-chronic exposure models has demonstrated that MC-LR administration induces varying degrees of histopathological damage across critical endocrine components, including the hypothalamus, pituitary, adrenal glands, testes, and thyroid [44]. Beyond direct gonadal damage, MCs mimic or antagonize endogenous hormones, leading to systemic endocrine dysfunction that can result in adverse reproductive outcomes such as subfertility and developmental delays [130].

MC-LR exerts endocrine-disrupting effects by directly targeting hypothalamic GnRH neurons, thereby impairing hormone synthesis and release upstream of the pituitary and gonads [40]. This neuroendocrine mode of action represents a distinct and underappreciated pathway of MC-LR-induced reproductive toxicity. The HPG axis comprises an integrated network of neural and endocrine tissues that coordinate reproductive function and fertility through tightly regulated hormonal signaling. A critical mechanism of this disruption is the induction of a blunted estrogen-negative feedback response, where the HPG axis fails to properly adjust gonadotropin secretion in response to fluctuating steroid levels [90]. In GnRH neurons, MC-LR triggers an influx of intracellular Ca^2+^ and cAMP, which activates the PKC, PKA, and MAPK signaling cascades [74,131]. While this initially stimulates hormone release, it ultimately suppresses *Gnrh1* gene transcription. At the transcriptional level, HPG axis disruption involves the activation of the cAMP/PKA/CREB/c-Fos signaling pathway, thereby repressing hormone transcription [73]. This activation subsequently downregulates key transcription factors such as Oct-1, Otx-2, and Pbx1a, while upregulating c-Fos and c-Jun, thereby repressing GnRH synthesis.

The molecular mechanism underlying MC-LR-induced male reproductive dysfunction involves the transcriptional downregulation of key steroidogenic markers, alongside reduced protein expression of steroidogenic acute regulatory protein (StAR) in the testes [44]. Importantly, these effects do not appear to result from direct agonistic or antagonistic interactions with the androgen receptor. Instead, accumulating evidence indicates that MC-LR perturbs endocrine regulation through indirect mechanisms, potentially involving altered hypothalamic–pituitary feedback signaling and toxin-induced modulation of steroid hormone metabolism [132]. Notably, MC-LR exerts distinct endocrine effects in female reproductive cells. By inducing StAR overexpression and progesterone overproduction in ovarian granulosa cells, MC-LR acts as an endocrine disruptor capable of triggering premature luteinization, a pathological condition known to impair endometrial receptivity and female fertility [83]. These findings suggest that MC-LR-induced endocrine disruption may have downstream consequences on implantation success and early pregnancy maintenance. In addition to disrupting steroidogenic regulation, MC-LR has also been proposed to exert estrogen-like activity. Owing to its estrogenic activity, MC-LR has been proposed as an environmental estrogen capable of activating estrogen receptor-mediated signaling pathways [133]. In the developing prostate, MC-LR disrupts the endocrine balance by upregulating estrogen receptor alpha (ERα) expression and interfering with the androgen-dependent signaling pathways [99]. Such receptor-level imbalances during critical developmental windows may contribute to abnormal prostate differentiation and long-term reproductive dysfunction. Beyond the gonads and accessory reproductive organs, placental endocrine function also appears to be a sensitive target of MC-LR exposure. The endocrine-disrupting effects of MC-LR in the placenta, such as altered hCG secretion, may be mediated by the toxin’s cellular uptake through OATP1B3, which is expressed in both cytotrophoblasts and syncytiotrophoblasts [97]. This disruption of placental hormone signaling highlights an additional mechanism by which MC-LR may disrupt pregnancy maintenance and fetal development.

## 6. Future Perspectives

Future research should prioritize environmentally relevant exposure models and longitudinal epidemiological studies. Recent human studies reporting associations between semen MC levels and impaired sperm quality highlight the urgent need for public health strategies to mitigate environmental exposure in school-aged children and adult populations [134]. Importantly, these findings suggest that reproductive effects may occur at environmentally relevant exposure levels, bridging the gap between animal toxicology and real-world human risk. Human exposure to MCs occurs primarily via three routes: direct dermal contact with contaminated water, oral ingestion through drinking water or food, and inhalation of aerosolized cyanotoxins [6,7]. This concern is particularly pronounced for children and adolescents, whose reproductive and endocrine systems remain highly sensitive during critical developmental windows. Accordingly, incorporation of reproductive and developmental endpoints into environmental monitoring and risk assessment frameworks constitutes a key priority for future research and policy development. Monitoring should focus on sensitive life stages, including pregnancy, early development, puberty, and reproductive adulthood. Priority endpoints include reproductive hormone profiles, gamete quality, placental function, fetal growth, and postnatal reproductive development.

A second priority is to resolve cellular heterogeneity within reproductive and placental tissues. Although MC-LR–induced gonadal toxicity is now comparatively well characterized, substantial knowledge gaps remain regarding non-gonadal reproductive organs [135]. In particular, the placenta plays central roles in hormonal integration, pregnancy maintenance, and developmental programming, yet its vulnerability to MC-LR exposure remains insufficiently defined. Addressing these gaps is essential for a more comprehensive understanding of reproductive risk across the entire reproductive axis. The placenta comprises highly heterogeneous cell populations with distinct endocrine, immune, and barrier functions, making them particularly susceptible to context-dependent toxic insults. To overcome these limitations, future research should increasingly employ emerging omics-based approaches, including single-cell RNA sequencing and spatial transcriptomics [136]. These technologies enable high-resolution dissection of MC-LR-induced molecular perturbations at the level of individual cell populations, while preserving spatial organization and developmental context. Future studies should combine single-cell transcriptomics with epigenomic profiling in matched reproductive tissues. Spatial transcriptomics can then locate vulnerable cell states within their tissue niches, while proteomic analysis can determine whether transcriptional changes lead to altered cellular pathways. Integrating these datasets with histological and reproductive outcomes would help distinguish primary cell-specific responses from secondary tissue damage.

Future studies should address the reproductive toxicity of co-occurring cyanotoxins and other environmental contaminants. While MC-LR has been the most intensively studied cyanotoxin with respect to reproductive and developmental toxicity, growing evidence indicates that other MC congeners and structurally distinct cyanotoxins may also pose risks to reproductive health and early development. Compounds such as cylindrospermopsin, β-N-methylamino-L-alanine (BMAA), and saxitoxins have been shown to exert systemic toxicity, cross physiological barriers, and induce developmental or neurodevelopmental defects under specific exposure conditions [137,138,139,140,141,142]. Importantly, comparative studies suggest that cyanotoxin-induced reproductive and developmental toxicity is highly heterogeneous, with toxin-specific targets, temporal sensitivity, and mechanistic pathways. Despite these observations, mechanistic insights into how non-MC-LR cyanotoxins affect reproductive tissues, placental function, and offspring development remain fragmented and insufficiently integrated. Future research should therefore move beyond a single-toxin paradigm and systematically evaluate the toxicity of diverse cyanotoxins, including less-studied MC congeners, under environmentally relevant exposure scenarios. Such efforts are particularly important given the frequent co-occurrence of multiple cyanotoxins in eutrophic waters, raising the possibility of additive or synergistic effects that are not captured by current risk assessment frameworks. Recent findings demonstrate that MC-LR–induced male reproductive toxicity can be markedly exacerbated by nitrite co-exposure, with mitochondrial dysfunction and dysregulated mitophagy emerging as convergent pathological mechanisms [143]. At the same time, emerging studies suggest that certain natural compounds can attenuate MC-LR-induced reproductive toxicity, pointing to potential avenues for intervention [144,145]. Future mixture studies should reproduce concentration ratios measured in bloom-affected water or food and apply repeated low-dose exposure across sensitive life stages. Resveratrol and icariin are among the better-characterized preclinical protective compounds, although current evidence is insufficient to compare their efficacy or support clinical use [108,145]. At the population level, strengthened toxin monitoring, bloom advisories, and temporary access to uncontaminated water could reduce exposure in communities dependent on affected water sources. The monitoring and regulatory implications proposed here are derived from mammalian evidence of chronic reproductive toxicity and emerging human evidence of internal exposure and impaired sperm quality. These implications identify priorities for future risk assessment, but the current evidence remains insufficient to support direct revision of regulatory standards.

## 7. Conclusions

Available mammalian evidence identifies the reproductive and developmental systems as important targets of MC-LR, although the strength of evidence remains uneven. Reproductive toxicity reflects both dose and cumulative exposure. Short-term high-dose administration primarily produces rapid cytotoxicity and overt gonadal injury. Repeated oral exposure at low μg/L concentrations instead causes progressive impairment of sperm quality, reproductive tissue architecture, and endocrine regulation over several months. These exposure categories are descriptive and do not constitute formal dose strata because the studies differ in species, route, and endpoint. Whether reproductive effects occur below the hepatotoxicity threshold remains unresolved. Immediate priority should be given to establishing environmentally relevant exposure models and life-stage-specific endpoints. Building on this foundation, cell- and tissue-resolved approaches can clarify mechanisms and support evidence-based risk guidelines.

## Figures and Tables

**Table 1:** Representative recent studies on MC-LR–induced male reproductive toxicity in mammals.

Experimental System	Species	Model	Exposure	Main Outcomes	Mechanisms/Pathways	References
In vitro	*Mus* *musculus*	GC-1 cells (Spermatogonia)	3, 6, 12 µg/mL for 24 h	Induction of DNA damage and cellular autophagy	Oxidative stress-mediated DNA damage and ATM/p53 signaling activation	[34]
		TM4 cells (Mouse Sertoli cells)	1, 2.5, 5, 7.5, 10 µg/mL for 24 h	Decreased expression of tight junction and adherens junction proteins	RhoA/ROCK activation; cytoskeleton disruption	[35]
		TM4 cells (Mouse Sertoli cells)	10, 20, 40 nM for 24 h	Induction of cell apoptosis and mitochondrial dysfunction	Dysregulation of intracellular Ca^2+^ homeostasis; activation of CaMKII and MAPK signaling; induction of ER stress	[36]
		GC-1 cells (Mouse spermatogonia)	0.2–5 μM for up to 48 h	Inhibition of cell proliferation; G2/M phase cell cycle arrest; excessive ROS production	Proteasomal degradation of SIRT6 via destruction of SIRT6-USP10 interaction; reduced PARylation activity of PARP1; impaired DNA repair.	[37]
		GC-2spd cells (Mouse spermatocytes)	100, 500 nM for 24 h	Reduced cell viability; induction of apoptosis	Direct binding of MC-LR to the catalytic domain of UCHL3; excessive GOPC ubiquitination and proteasomal destruction	[38]
		TM3 cells (Mouse Leydig cells)	0.3125–5 μM for 24 h	Reduced expression of steroidogenic proteins and synthases	Activation of the ROS-mediated GCN2/eIF2α signaling pathway	[39]
		GT1-7 cells (Mouse hypothalamic GnRH neurons)	50, 500, 1000 nM for 24 h	Reduced cell viability; induction of apoptosis	Activation of ER stress; induction of protective autophagy; ROS and Ca^2+^ signaling involvement	[40]
	*Homo* *sapiens*	RWPE-1 cells (Human prostate epithelial)	10, 100, 500 nM for 25 weeks	Malignant transformation; enhanced colony formation, migration, and invasion	Upregulation of FOXM1 and COX-2 expression; induction of malignant phenotype	[41]
		DU145 cells (Human prostate cancer)	1, 5 μM for 24 h	Increased cell invasion capacity; microfilament rearrangement	Activation of ERK phosphorylation; enhanced phosphorylation of microfilament-associated proteins VASP and ezrin	[42]
In vivo	*Mus* *musculus*	C57BL/6N mice	10, 20, 40 µg/kg (i.p., 14 days)	Reduced gonadosomatic index; testicular pathological damage; sperm count reduction	ATM/p53 pathway activation and induction of autophagy	[34]
		C57BL/6J mice	1, 30, 60, 90, 120 µg/L in drinking water for 6, 9, and 12 months	Testis structure loss, germ cell abscission, and BTB damage.	Activation of the RhoA/ROCK pathway; rearrangement of α-tubulin	[35]
		Pubertal ICR mice	2 μg/kg (i.p., 4 weeks)	Decreased epididymal sperm concentration; blocked spermatogonia proliferation	Oxidative DNA damage; inhibition of SIRT6-mediated DNA repair.	[37]
		BALB/c mice	1, 10, 20, 30 μg/L in drinking water for 180 days	Disruption of acrosome biogenesis; pathological changes in seminiferous tubules; selective reduction of spermatocytes and round spermatids	Disruption of the UCHL3-GOPC regulatory axis; proteasomal degradation of GOPC	[38]
		BALB/c mice	1, 7.5, 15, 30 μg/L in drinking water for 180 days	Induction of prostatic intraepithelial neoplasia and microinvasion; increased angiogenesis in prostate tissues	Activation of the FOXM1/COX-2 signaling pathway	[41]
		BALB/c nude mice	40 μg/kg (oral gavage, 4 weeks)	Promotion of tumor local invasion	Activation of the ERK/VASP/ezrin signaling pathway	[42]
		ICR mice	20 μg/kg (i.p., daily for 5 weeks)	Decreased serum and testicular testosterone levels	Downregulation of steroidogenic proteins (StAR, CYP11A1, CYP17A1) in testes	[39]
		BALB/c mice	1, 7.5, 15, 30 μg/L in drinking water for 180 days	Apoptosis of GnRH neurons in the hypothalamus; decreased serum testosterone levels	Activation of ER stress and autophagy	[40]
	*Rattus norvegicus*	Sprague-Dawley rats	Single i.p. injection of 45, 67.5, or 90 μg/kg (24 h)	Histological damage in the hypothalamus, pituitary, and testis; decreased serum GnRH and testosterone levels; increased serum LH, FSH, and estradiol levels	Disruption of the HPG axis; imbalance of hormonal feedback loops; downregulation of steroidogenic enzymes and genes (e.g., *StAR*, *3β-HSD*, *Cyp11a1*)	[43]
		Sprague-Dawley rats	3, 30 μg/kg (i.p., daily for 6 weeks)	Histological damage to the hypothalamus, pituitary, and testis; decreased serum GnRH and DHT; increased serum LH, FSH, T, and E2	Disruption of the HPG axis; alteration of hormone synthesis and secretion (e.g., *StAR*, *3β-HSD*, *Cyp11a1*, *Cyp17a1*)	[44]

**Table 2:** Representative recent studies on MC-LR–induced female reproductive toxicity in mammals.

Experimental System	Species	Model	Exposure	Main Outcomes	Mechanisms/Pathways	References
In vitro	*Mus musculus*	KK-1 cells (Mouse ovarian granulosa cells)	8.5, 17, 34 μg/mL for 24 h	Reduced mitochondrial membrane potential; increased apoptosis rate	Oxidative stress-induced ASK1 expression; activation of P38/JNK signaling pathway	[77]
		Mouse granulosa cells	4.25, 8.5, 17 μg/mL for 24 h	Reduced cell viability; induction of apoptosis; increased expression of pro-apoptotic factors	ER stress-mediated CHOP activation; upregulation of Dr5 and active-caspase-3	[79]
		Mouse primary granulosa cells	0.01, 0.1, 1 μM for 24 h	Mitochondrial fragmentation; decreased glucose intake; mitochondrial dysfunction	Activation of ROS/FOXM1/DRP1 pathway; downregulation of GLUT1 and GLUT4	[84]
		Mouse ovarian follicles	0.1, 1, 10 μM for 4 days	Dose-dependent inhibition of follicle rupture and oocyte meiosis; reduced progesterone secretion	Selective inhibition of PP1 activity; disruption of PI3K/AKT/FOXO1 signaling	[85]
		Mouse endometrial stromal cells	1 μM for 24–48 h	Inhibited cell differentiation; decreased expression of decidual markers	Inhibition of PP2A activity; disruption of the PI3K/AKT/FOXO1 axis	[86]
	*Homo sapiens*	KGN cells (Human ovarian granulosa)	0.01, 0.1, 1 μM for 24 h	Inhibition of glycolysis	Inhibition of AKT phosphorylation and activation of GSK3β; HK2 dissociation from the mitochondrial membrane	[80]
		Human primary granulosa cells	10 μM for 24 h	Reduced expression of follicle maturation genes	Inhibition of PP1 activity; reduced phosphorylation of FOXO1	[85]
		HUVECs	0.01, 0.1, 1 μM for 24 h	Inhibited migration and tube-forming capacity	Suppression of the MEK/ERK/SP1/VEGFR2 signaling axis	[87]
		HTR-8/SVneo (Human trophoblast)	1 μM for 24 h	Increased secretion of trophoblast-derived extracellular vesicles containing miR-377-3p	Inhibition of NR6A1; activation of the mTOR/S6K1/SREBP signaling pathway	[88]
		HTR-8/SVneo (Human trophoblast)	1, 2, 3 μM for 24 h	Decreased cell migration and invasiveness	Down-regulation of the AKT/mTOR/HIF-1α signaling pathway	[89]
In vivo	*Mus musculus*	C57BL/6 mice	40 μg/kg (i.p., daily for 2 weeks)	Ovarian pathological damage; increased ovarian apoptosis; decreased gonadosomatic index	Activation of the ASK1-mediated P38/JNK pathway via oxidative stress; activation of Fas-mediated death receptor and P53-mediated mitochondrial pathways	[77]
		C57BL/6 mice	40 μg/kg (i.p., daily for 14 days)	Induction of ovarian apoptosis, inflammation, and follicular atresia; decrease of gonadosomatic index	Activation of ER stress; upregulation of CHOP; inhibition of PP2A activity	[79]
		BALB/c mice	10, 20 μg/kg (i.p., daily for 14 days)	Ovarian morphological damage; hormonal imbalance (decreased E2, P4; increased FSH, LH); decreased number of healthy follicles and increased atretic follicles	Disruption of ovarian glycolysis	[80]
		CD-1 mice	10 μg/kg (p.o., daily for 6 wk)	Significant reduction in the number of corpora lutea	Interference with gonadotropin-dependent follicle maturation; inhibited ovulation	[85]
		Pregnant BALB/c mice	15 μg/kg (i.p., daily for 6–7 days)	Reduced embryo implantation sites and embryo volume; inhibited estrogen and progesterone synthesis	Impairment of luteal angiogenesis; disruption of the vascular network in the corpus luteum	[87]
		Pregnant BALB/c mice	40, 200 μg/kg (p.o., daily from GD1 to GD18)	Abnormal M1 polarization of placental macrophages; induced placental inflammation	Alteration of miRNA profiles in trophoblast-derived extracellular vesicles	[88]
		Pregnant BALB/c mice	40, 200 μg/kg (p.o., daily from GD1 to GD18)	Preeclampsia-like symptoms; vascular dysplasia in the placenta	Inhibition of VEGFA and TGFβ expression	[89]
		Pregnant ICR mice	15, 30 μg/kg (i.p., daily from GD1 to GD8)	Reduced uterine weight and number/weight of implantation sites; impaired endometrial decidualization	Suppression of the PI3K/AKT/FOXO1 signaling pathway	[86]
	*Rattus norvegicus*	Wistar rats	5, 20, 40 μg/kg (i.p., single dose)	Disruption of the HPG axis; reduced LH and estradiol levels; increased FSH, progesterone, and AMH; reduced primary follicles	Impairment of the HPG axis; ovarian oxidative stress and inflammation; blunted estrogen-negative feedback	[90]

**Table 3:** Studies on developmental outcomes in offspring following MC-LR exposure.

Experimental System	Species	Model	Exposure Window	Dosage & Route	Affected Organ	Main Findings	References
In vivo	*Mus musculus*	C57BL/6 mice	Pregestational and gestational (12 weeks + GD1–Birth)	15, 30 μg/L (drinking water)	Pancreas	Lower blood and plasma glucose levels; higher fasting serum insulin levels; suppressed pancreatic β-cell proliferation	[103]
		BALB/c mice	Prenatal (GD1–18)	40, 200 μg/kg (p.o., daily)	Heart	Thinner myocardial walls; enlarged ventricles; impaired cardiac systolic function	[88]
		BALB/c mice	Perinatal (GD12–PND21)	1, 10, 50 µg/L (drinking water)	Prostate	MC-LR accumulation; decreased prostate index; impaired prostate development; decreased serum E2 levels (30 days); reduced serum T and T/E2 ratio (90 days)	[99]
		BALB/c mice	Perinatal (GD12–PND21)	10, 50 µg/L (drinking water)	Prostate	Induced prostatic epithelial hyperplasia	[100]
		BALB/c mice	Perinatal (GD12–PND21)	10 µg/L (drinking water)	Testis	Decreased testis index; damaged testicular structure; increased cell apoptosis, decreased cell proliferation	[47]
		BALB/c mice	Paternal exposure (6 months)	1, 7.5, 15, 30 µg/L (drinking water)	Lung	Impaired lung development; thickened alveolar walls; collagen deposition	[104]
	*Rattus norvegicus*	Sprague-Dawley rats	Perinatal (GD8–PND15)	10 µg/kg, s.c.	Brain	MC-LR accumulation; mitochondrial swelling; ER distention; sparse tissue architecture; oxidative stress	[101]
		Sprague-Dawley rats	Perinatal (GD8–PND15)	10 µg/kg, i.p	Liver	MC-LR accumulation; widened intercellular spaces; fat droplets, oxidative stress	[102]

## Data Availability

No new data were created or analyzed in this study. Data sharing is not applicable to this article.

## Supplementary Materials

The following supporting information can be downloaded at https://www.mdpi.com/article/10.3390/toxics14080734/s1, Figure S1: Two-dimensional chemical structure of microcystin-LR (MC-LR). MC-LR is a cyclic heptapeptide containing L-leucine and L-arginine at the variable X and Y positions, respectively, and the characteristic Adda moiety. The structure image was obtained from PubChem (CID 445434); Figure S2: Literature search and study selection process.

## Abbreviations

The following abbreviations are used in this manuscript:

MC-LR	Microcystin-LR
HPG	Hypothalamic-pituitary-gonadal
OATP	Organic anion transporting polypeptide
BTB	Blood-testis barrier
ER	Endoplasmic reticulum
hCG	Human chorionic gonadotropin
GD	Gestational day
PND	Postnatal day
ROS	Reactive oxygen species
GSH	Glutathione
GPx	Glutathione peroxidase
SOD	Superoxide dismutase
NAC	N-acetyl-L-cysteine
DRP1	Dynamin-related protein 1
CaMKII	Calmodulin-dependent protein kinase II
PARP1	Poly(ADP-ribose) polymerase 1
miRNA	microRNA
piRNA	PIWI-interacting RNA
circRNA	circular RNA
lncRNA	long non-coding RNA
UTR	untranslated region
PKA	protein kinase A
CDK6	cyclin-dependent kinase 6
EDC	endocrine-disrupting chemical
StAR	steroidogenic acute regulatory protein
BMAA	β-N-methylamino-L-alanine
