# Supplementary material for: Reproductive and Developmental Toxicity of Microcystin-LR in Mammals
Source: Toxics. 2026 Aug 21;14(8):734. doi: 10.3390/toxics14080734 (PMC13517335; doi:10.3390/toxics14080734)
Supplement: Supplementary file 1 [file toxics-14-00734-s001.zip › Figure S2.pdf]

Literature Search and Study Selection

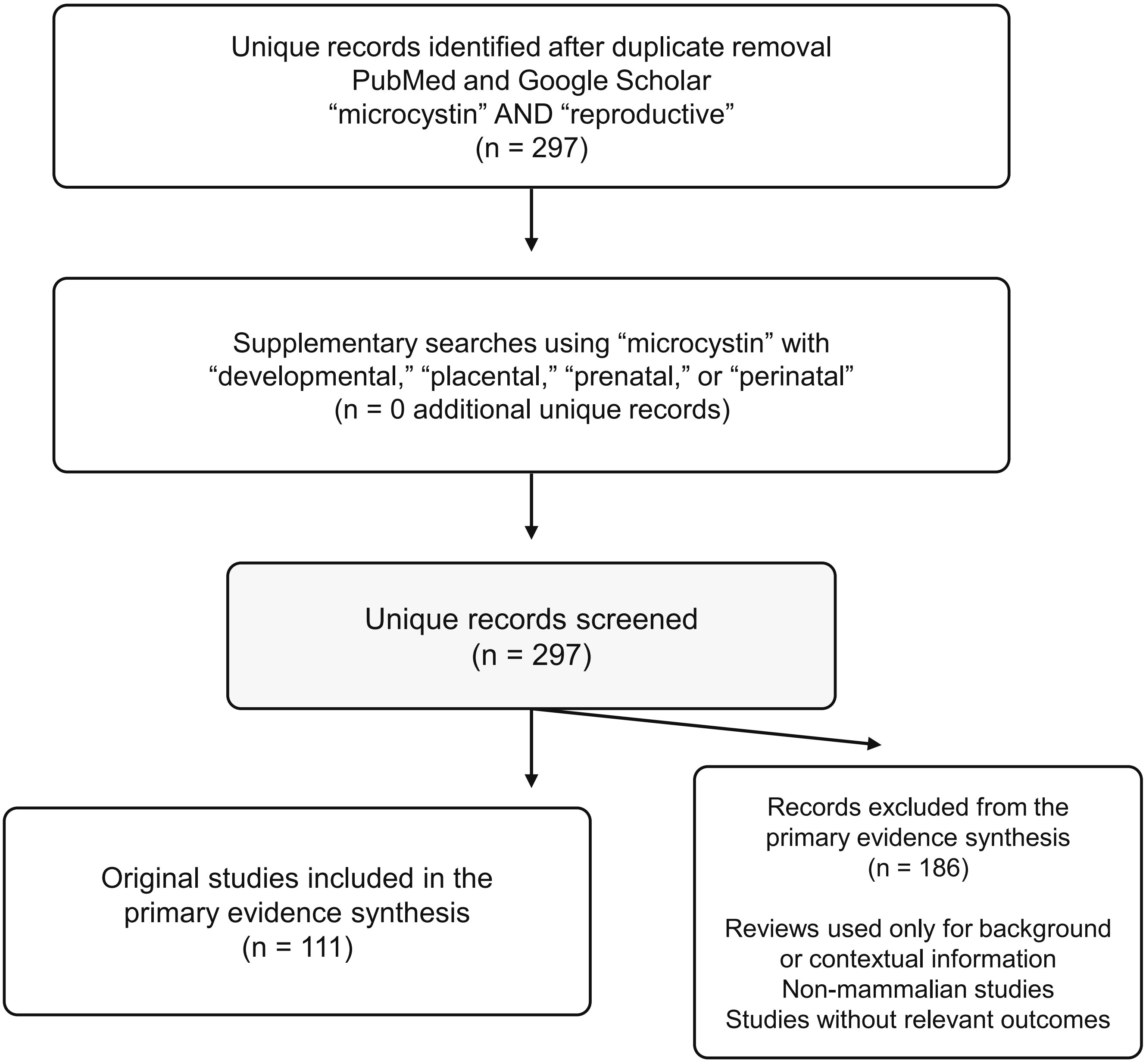

Search period: database inception to 31 January 2026
